# La_0.6_Ca_0.4_CoO_3_ Perovskite Nanofibers by Electrospinning: Preparation and Properties

**DOI:** 10.1021/acsomega.5c13104

**Published:** 2026-05-19

**Authors:** Onur Alp Aksan, Esin Cagla Konukcu, Mehmet Sezer, Leyla Çolakerol Arslan, Nuray Kizildag

**Affiliations:** † Institute of Nanotechnology, 52962 Gebze Technical University, Kocaeli 41400, Turkey; ‡ Department of Materials Science and Engineering, Gebze Technical University, Kocaeli 41400, Turkey; § Department of Physics, Gebze Technical University, Kocaeli 41400, Turkey

## Abstract

Perovskite nanofibers offer a unique combination of structural and functional advantages by uniting the high-surface area, flexibility, and directional geometry of nanofibers with the exceptional optoelectronic and catalytic properties of perovskite materials. In this work, La_0.6_Ca_0.4_CoO_3_ (LCC) perovskite nanofibers—whose synthesis has not previously been reported in the literature—were successfully synthesized via electrospinning. Polyacrylonitrile (PAN) was used as the carrier polymer, and an optimization identified an electrospinning solution containing 7 wt % PAN and LCC precursors at a PAN/LCC ratio of 1:1 as the ideal composition. The nanofibers fixed onto a ceramic pellet and calcined at 700 °C exhibited a uniform and continuous one-dimensional morphology with an average fiber diameter of 578 nm. Stable LCC phase formation was confirmed by FTIR, TGA, and XRD analyses, and phase identification further verified that the calcined nanofibers crystallized into a rhombohedral perovskite structure. XPS analysis revealed that surface oxygen vacancy concentrations varied with calcination temperature, and significant segregation occurred at elevated temperatures. According to the BET measurements, the specific surface area of the nanofibers increased along with pore size and pore volume upon calcination. Overall, these results demonstrate that LCC perovskite nanofibers with high-surface area, nanoscale diameter, one-dimensional architecture, and tunable oxygen vacancy concentrations can be obtained, highlighting their strong potential for applications in energy conversion, gas sensing, catalysis, and intermediate-temperature solid oxide fuel cells.

## Graphical abstract

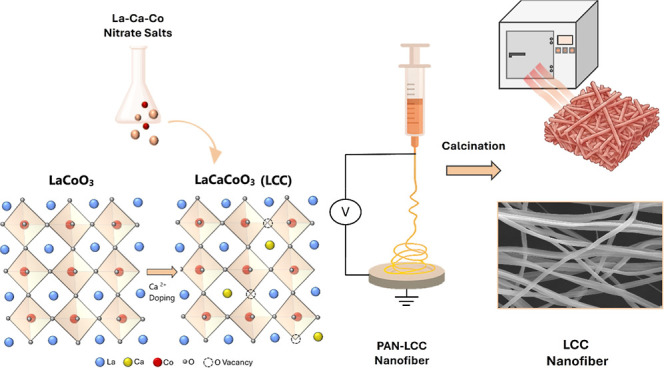

## Introduction

Ceramic materials are widely recognized for their outstanding properties, including excellent corrosion resistance and high tolerance to extreme temperatures and valuable applications in semiconductors. With the growing demand for advanced, high-performance materials and continuous miniaturization, attention has shifted toward understanding ceramics’ unique behaviors at nanoscale. Depending on the intended application, ceramic materials can be synthesized in various geometries and dimensions, including nanoparticles (0D), nanofibers (1D), thin films (2D), and bulk structures (3D).^1^

Ceramic nanofibers are particularly noteworthy, typically featuring an outer diameter of less than 1000 nm and a length-to-diameter (L/D) ratio greater than 50. These structures retain the exceptional properties of ceramics while offering the benefits of nanomaterials.^2^ Electrospinning is emerging as the most versatile and flexible method for producing ceramic nanofibers. The process of creating ceramic nanofibers through electrospinning involves three main steps: preparing an electrospinning solution that includes precursor salts, a carrier polymer, and a solvent; electrospinning to produce the nanofibers; and calcination. To perform electrospinning, it is essential to combine the precursor salts, carrier polymer, and solvent in the correct proportions to create a homogeneous solution with an appropriate viscosity.^1–6^ The characteristics of ceramic nanofibers can be controlled to a great extent by altering the parameters such as precursor composition, spinning parameters, solution feed rate, calcination temperature, and time.^7–10^ Numerous investigations have revealed that alteration of parameters like polymer concentration, solution viscosity, applied voltage, and tip-to-collector distance can result in substantial variations in both fiber morphology and crystal structure. This broad range of tunable processing conditions not only increases the usage areas by changing the nanofiber properties such as size, diameter, and geometry of the nanofibers to be produced but also provides great opportunities to optimize them for specific application areas.^11–17^ Their distinct characteristics, such as high porosity, large surface area, and excellent thermal and chemical stability, make ceramic nanofibers highly versatile for a wide range of applications, from water remediation^18,19^ and sensors^6,20^ to energy^21,22^ and biomedical uses.^7,23–27^

Perovskite oxides, with the general formula ABO_3_, constitute a versatile class of materials where A is a larger cation (such as La^3+^, Sr^2+^, or Ba^2+^) and B is a smaller transition metal cation (like Ti^4+^, Fe^3+^, or Mn^3+^), surrounded by an octahedral network of oxygen atoms. This flexible crystal structure allows for extensive chemical substitution, enabling fine-tuning of their electrical, magnetic, optical, and catalytic properties. As a result, ABO_3_ perovskites exhibit a wide range of functional behaviors, including ferroelectricity, superconductivity, magnetoresistance, and high ionic conductivity. Oxygen vacancies (V_o_) in perovskites play a crucial role in enhancing their performance across a wide range of applications. Because perovskites are mixed ionic–electronic conductors, introducing oxygen vacancies can dramatically alter their electrical conductivity, catalytic activity, and surface reactivity. For gas sensing applications, vacancies act as adsorption and reaction sites for target gas molecules (O_2_, NO_2_, CO, H_2_, etc.) and cause changes in resistance or photoluminescence when gases interact with the surface, improving sensitivity. In solid oxide electrolytes and cathodes, they enable oxygen ion transport through the lattice. For photocatalysis applications, oxygen vacancies create defect states in the band gap, enhancing visible-light absorption, they act as trap sites that reduce electron–hole recombination and promote surface redox reactions by facilitating charge transfer.^28^ Also, controlled vacancies help balance ionic and electronic transport, improving thermoelectric performance.^29^ Perovskite nanofibers combine the unique optoelectronic and catalytic properties of perovskite materials with the high-surface area, flexibility, and anisotropic structure of nanofibers.

LaCoO_3_ (LCO) is a perovskite oxide with the ABO_3_ structure, where La^3+^ occupies the A-site and Co^3+^ the B-site, forming a stable rhombohedral lattice. It exhibits moderate electronic conductivity, limited oxygen ion mobility, and good catalytic activity due to the redox flexibility of Co^3+^ ions, making it suitable for use as a cathode in solid oxide fuel cells (SOFCs), as well as in gas sensors and oxidation catalysts.^30^ Murai et al. (2018) investigated LaCoO_3_ as a thermoelectric material and how Ca and Fe doping affected the thermoelectric properties.^31^ Ateia et al. (2019) prepared LaCoO_3_ mesopores sensors by a modified citrate technique in order to measure and control humidity. The prepared LaCoO_3_ exhibited large surface area of 154 cm^2^ g^–1^, good crystallinity, and small crystallite size, which made it suitable for humidity sensor application. The impedance was reduced by approximately eight times its normal magnitude with increasing the humidity level.^32^ Wang et al. (2022) investigated the Ce, Ca, Fe, and Mn-doped LaCoO_3_ perovskite oxides prepared via the sol–gel method for the four-way purification of particulate matter (PM), nitrogen oxides (NO_*x*_), carbon monoxide (CO), and hydrocarbons (HC) from diesel engine exhaust. La_0.8_Ce_0.2_Co_0.7_Fe_0.3_O_3_ perovskite displayed the best purification efficiencies of 95%, 92%, 94%, and 100% for PM, NO_*x*_, CO, and HC, respectively.^33^ Moorthi et al. (2024) prepared perovskite-structured lanthanum cobalt oxide (LaCoO_3_/LCO) systems with particle and flake morphologies using sol–gel and hydrothermal methods, respectively, in order to investigate their morphological structure-dependent properties for potential supercapacitor applications. They suggested that the properties of perovskite LaCoO_3_ could be tuned by adjusting its morphology through various synthesis methods, making LaCoO_3_ a viable and robust system for energy storage applications.^34^ Sun et al. (2025) synthesized La_1–*x*_Ca_*x*_CoO_3_ (*x* = 0, 0.1, 0.15, and 0.2) nanoparticles using the sol–gel method and studied the impact of Ca ions substitution at A-site on performance of LaCoO_3_ perovskites for energy applications. The La_0.85_Ca_0.15_CoO_3_ sample exhibited a specific capacitance of 284.4 F g^–1^ at a current density of 1 A g^–1^, which was 3.38 times greater than that of the intrinsic sample. The charge storage mechanism involving the oxygen anions was explored through an overcharge and discharge process, revealing that elemental Ca doping markedly enhanced the charge storage capacity associated with oxygen intercalation in the LaCoO_3_ system. The results obtained suggested that Ca-substituted A-site of perovskites possessed significant potential for supercapacitor applications.^35^ Sezer et al. (2020) prepared polymeric precursor-derived films of LaCoO_3_ doped with Ca^2+^ (LCC), instead of the larger Sr^2+^ which tends to segregate at the electrode surface forming oxides/hydroxides and investigated a correlation between the phase, microstructure, and surface chemistry evolution and electrochemical performance.^36^

Some studies exist in literature reporting on the fabrication of LaCoO_3_ nanofibers (NFs) and their applications in different areas. Wang et al. (2010) prepared crystalline LaCoO_3_ NFs by calcination of the PVA/[La­(NO_3_)_3_+Co­(CH_3_COO)_2_] composite nanofibers at 600 °C for 10 h. The diameter of LaCoO_3_ NFs was ca. 80 nm, and the length was greater than 100 μm. The polycrystalline LaCoO_3_ NFs with single phase were synthesized when calcination temperature was in the range of 600–900 °C. The crystal structure of the prepared LaCoO_3_ was trigonal system with space group *R*3*m*.^37^ Dong et al. (2010) fabricated LaCoO_3_ NFs by electrospinning and subsequent calcination technology and investigated their applicability as catalysts for photocatalytic degradation of Rhodamine B. Results showed that the samples calcined at 600 °C exhibited the best photocatalytic activity at pH.^38^ Shim et al. (2015) prepared LaCoO_3_ NFs through the calcination of an electrospun polymer–metal precursor fiber and investigated the electrochemical performance of these fibers for oxygen reduction and evolution reactions in a KOH solution in comparison to conventional PtRu/C catalyst and a LaCoO_3_ powder. The LaCoO_3_ NFs had a greater surface area compared with the powder, whereas the crystal structures of the fibers and powder were notably similar. The LaCoO_3_ NFs demonstrated better electrochemical properties compared with the LaCoO_3_ powder, which was attributed to the increased surface area and number of active sites in the fibers.^39^ Shingange et al. (2020) reported on the fabrication of remarkably selective and sensitive LaCoO_3_ NF-based sensors obtained after annealing at different temperatures of 550, 650, and 700 °C. Systematic gas sensing analysis revealed that the sensors based on LaCoO_3_ NFs have substantial sensitivity to 40 ppm ethanol gas with the sensor obtained at 650 °C revealing an outstanding response of 32.4 at a lower optimum operating temperature of 120 °C, which stemmed from the nanofibrous structure, providing high surface and porous channels.^40^ Recently, some researchers tailored the structure of LCC perovskite by A-site and/or B-site doping for enhanced performance in different applications. Wahed et al. (2022) prepared single-phase La_1–*x*_Sr_*x*_CoO_3_ (LSC, *x* = 0, 0.01, 0.03, 0.05, 0.07, and 0.1) NFs and reported that LSC NFs exhibited semiconductor-like behavior. The electrical conductivity of all the samples was found to decrease with increasing concentration of Sr doping, whereas the Seebeck coefficient of all the fabricated devices was found to increase.^41^ Guo et al. (2024) fabricated La_1–*x*_Sr_*x*_Co_1‑δ_Fe_δ_O_3_ (LSCF) hollow NFs via the electrospinning method. The hollow nanofiber structure was constructed by doping Sr atoms in the A-site and Fe atoms in the B-site of LaCoO_3_ perovskite. The characterization of TEM proved that the hollow tubular structural LSCF NF exhibited an external diameter of about 95 nm and an internal diameter of about 50 nm. The hollow tubular structural LSCF NF showed superior carrier separation ability which could exhibit significant advantages for photocatalytic CO_2_ reduction.^42^ Guo et al. (2025) performed a systematic investigation to verify the effects of Sr doping levels on the morphology, microstructure, and electrochemical properties of La_1–*x*_Sr_*x*_CoO_3_ (LSC, *x* = 0, 0.1, 0.15, 0.2) NFs and reported a maximum specific capacitance of 265.5 F g^–1^ at a current density of 1 A g^–1^ for La_0.85_Sr_0.15_CoO_3_ NFs, which showed the effectiveness of A-site Sr doping in enhancing the electrochemical properties of perovskite.^43^ Cao et al. (2025) prepared La_1–x_Ce_*x*_CoO_3−δ_ (*x* = 0, 0.05, 0.1, 0.15, and 0.2) perovskite NFs by tailoring the LaCoO_3_ perovskite oxides via Ce-substitution and electrospinning followed by calcination. As Ce substitution increased, the perovskite transitioned from a single hexagonal phase to a dual-phase (hexagonal and cubic) structure. Ce substitution was found to enhance the overall structural stability and oxygen vacancy concentration. Although the nanofiber structure was not well preserved after the calcination process, the improvement in the electrochemical and cycling performance of the supercapacitor device was attributed to the relatively intact nanofiber structure providing abundant active sites and the lowest internal resistance.^44^

Building upon the current state of the art, this study aims to develop uniform and continuous La_*x*_Ca_1–*x*_CoO_3_ (LCC) perovskite nanofibers, which are reported here for the first time. Previous studies have demonstrated that LaCoO_3_ (LCO) materials in various forms, including particles, flakes, nanoparticles, and thin films, have been explored for a wide range of applications (gas sensing, catalysts, thermoelectric devices, energy applications, etc.). It has been consistently reported that doped LCO systems exhibit enhanced functional performance compared to undoped compositions. In particular, the partial substitution of La^3+^ with Ca^2+^ to form LCC introduces Co^4+^ ions and oxygen vacancies, which enhance electronic and ionic conductivity, improve catalytic activity toward oxygen reduction and evolution reactions, and induce ferromagnetic behavior through double–exchange interactions. Moreover, LCO nanofibers have been shown to outperform other morphologies due to their high-surface area and favorable structural characteristics. Building on these insights, the present work reports the first successful synthesis of Ca-doped LCO nanofibers and provides a systematic examination of their structural properties, as further performance improvements in various applications may be expected when LCC materials are prepared in the nanofiber form. The nanofibers are fabricated via an electrospinning technique, followed by calcination, and a novel strategy is proposed to preserve the continuous nanofiber morphology during the calcination process. The La/Ca ratio of 0.6:0.4 was intentionally selected as a representative high Ca-doping composition to facilitate a clearer investigation of composition-dependent structural behavior in the nanofiber system. The effects of polymer concentration, precursor concentration, and calcination temperature on the morphology and structural characteristics of both the as-spun composite and the resulting perovskite nanofibers were systematically investigated. In addition, variations in the oxygen vacancy concentration as a function of calcination temperature are examined and discussed. Overall, this work fills an important gap in the literature by establishing a novel and structurally stable Ca-doped perovskite nanofiber system, providing a solid foundation for future functional and application-oriented studies.

## Materials and Methods

### Materials

Polyacrylonitrile (PAN, average *M*_n_ ≈ 150,000 g·mol^–1^, Sigma-Aldrich) and *N*,*N*-Dimethylformamide (DMF, anhydrous, 99.8%, Sigma-Aldrich) were used as received without further purification. Lanthanum nitrate hexahydrate [La­(NO_3_)_3_·6H_2_O], calcium nitrate tetrahydrate [Ca­(NO_3_)_2_·4H_2_O], and cobalt nitrate hexahydrate [Co­(NO_3_)_2_·6H_2_O] were purchased from Sigma-Aldrich and used directly in precursor solution preparation.

### Solution Preparation

A feeding solution of PAN/DMF with polymer concentration ranging from 5% to 8% was prepared for the electrospinning process. The required amount of PAN was gradually added to DMF and continuously stirred at 60 °C for 3 h to ensure the formation of a homogeneous solution. After stirring, the prepared solutions were allowed to cool to room temperature before further processing. Following the acquisition of a consistent and uniform nanofiber structure from a PAN solution, the same PAN/DMF solution was prepared under identical conditions and heated at 60 °C. To obtain a ceramic fiber structure, the nitrate precursors of lanthanum, calcium, and cobalt were added to the previously prepared PAN/DMF solution at a fixed PAN: total salt weight ratio of 1:1, with the La and Ca nitrate salts introduced in a La: Ca molar ratio of 0.6:0.4. This single composition with a relatively high Ca content (La_0_._6_Ca_0_._4_CoO_3_) was intentionally selected to serve as a representative case in which dopant-induced segregation and defect-related phenomena are more pronounced. Since the tendency for cation segregation and defect formation generally increases with dopant concentration, the use of a higher Ca-doping level allowed us to more clearly capture and analyze nanofiber growth behavior, structural continuity, and morphology evolution under enhanced segregation driving forces. To ensure the homogeneous distribution of nitrate salts, the mixture was stirred for an additional 3 h at 60 °C. The final solution obtained was homogeneous, clear, and suitable for electrospinning (Figure 1).

**Figure 1:**
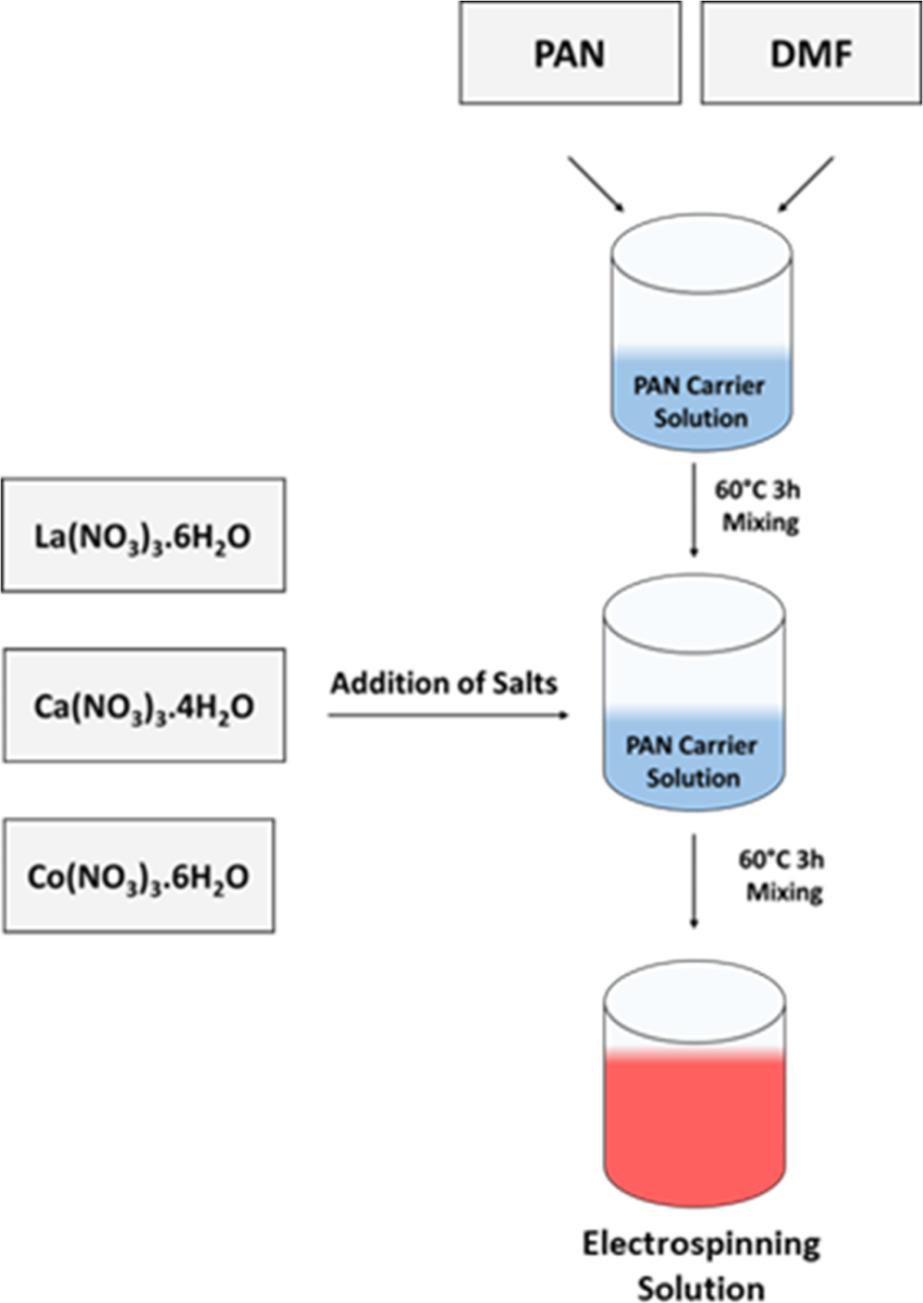
Flowchart for the preparation of electrospinning solution.

### Electrospinning

After the solution reached room temperature, electrospinning was carried out. Nanofibers were produced on a horizontal electrospinning apparatus that was equipped with a syringe pump (New Era, NE 1000), a high-voltage power supply (Inovenso, 0–30 kV), and a drum collector with homogenization (Inovenso, 0–500 rpm) (Figure 2). Electrospinning solutions were added to a 10 mL syringe with a blunt needle (Ultradent Products Inc., 18 Gauge, outer diameter: 1.25 mm) and fed with the flow rate of 3 mL/h toward the tip of the needle. A voltage of 25 kV was applied to the polymer solution to produce electrically charged jets that converted to nanofibers with the effect of the electrical field between the anode (needle) and the cathode (collector). The rotating cylindrical collector was placed at a distance of 11 cm from the tip of the needle, and the nanoweb was collected onto an aluminum foil which was wound around the drum collector. This optimized set of process parameters was established through a detailed and systematic optimization study and was deliberately selected to achieve an optimal balance among fiber uniformity, structural integrity after calcination, and practical material yield. The drum collector with a homogenization function ensured that the electrospinning process consistently produced homogeneous PAN and PAN-LCC nanofibers.

**Figure 2:**
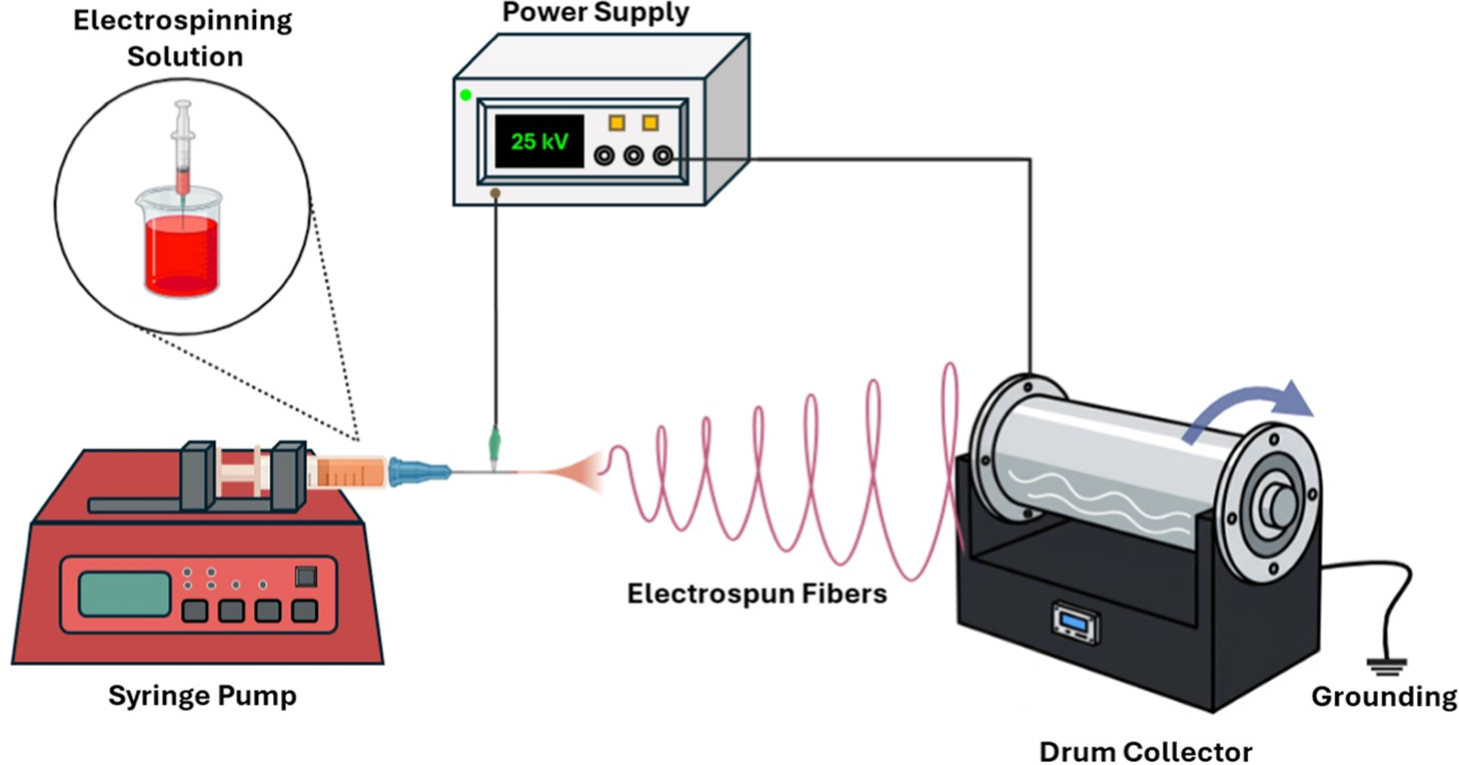
Schematic representation of the electrospinning process.

### Calcination of Nanofibers

A calcination procedure was applied to remove the polymeric carrier material and other organic species and promote the formation of the desired perovskite crystal structure as a result of which, LCC perovskite nanofibers were acquired from the PAN-LCC composite nanofibers. After the nanofiber samples were collected, they were put in an alumina crucible and heated to 700 °C, 800 °C, and 900 °C at a controlled heating rate of 4 °C/min and calcined at each target temperature for 3 h. After calcination, the furnace was turned off, and the samples were left to cool naturally to room temperature.

### Characterization

The viscosities of the electrospinning solutions were measured with a IKA viscometer using Elvas spindle. Hanna HI 8633 conductivity meter was used to measure the conductivity of the electrospinning solutions. Scanning electron microscopy (SEM) was used to take images of the nanofibers. The samples were sputter coated with gold layer before the analysis. The diameters of at least 50 randomly selected nanofibers were measured on SEM photomicrographs, which were taken with the same magnification, using Image Analysis Software and the average of the nanofiber diameter was calculated. Representative SEM images were selected to illustrate each nanofiber web. The average nanofiber diameters were expressed as the mean ± standard deviation. Wide-angle X-ray diffraction (XRD) traces of the nanowebs were obtained by the Rigaku SmartLab X-ray diffractometer with an operating voltage and a current setting of 40 kV and 30 mA, respectively, with utilization of nickel-filter and CuKα radiation (wavelength = 0.154056 nm). The diffraction patterns of the reference and composite nanofibers were obtained by scanning the samples in the 10–80° 2θ range with the rate of 1°/min. PAN, PAN-LCC, and LCC nanofibers obtained at different calcination temperatures were characterized with regard to their crystal structure. Their mean crystal size was calculated by the Scherrer equation. The Shimadzu IRSpirit-T model spectrometer was used to obtain the Fourier transform infrared (FTIR) absorption spectra of PAN, PAN-LCC, and LCC nanofibers. A minimum of 16 scans with a signal resolution of 4 cm^–1^ within the 400–4000 cm^–1^ range were averaged. TGA measurements were conducted using a METTLER TOLEDO TGA3+ in atmospheric conditions and within a temperature range of 25–1000 °C. X-ray photoelectron spectroscopy (XPS) measurements were performed on a Phoibos 150 XPS system (SPECS GmbH) utilizing a hemispherical electron analyzer and an Al Kα X-ray (*h*_v_ = 1486.6 eV) as the excitation source. The binding energies were calibrated by referencing the C 1s peak (284.6 eV). Spectral deconvolution was performed using a hybrid Gaussian–Lorentzian peak-fitting approach, and the background was removed using the Shirley algorithm. The concentrations of the individual components were then determined from the integrated peak areas after applying photoionization cross-section corrections. The Brunauer–Emmett–Teller (BET) method was adopted, and Micrometrics BET (Brunauer–Emmett–Teller -3Flex series) devices was utilized to determine the specific surface area and pore size of PAN, PAN-LCC, and LCC nanofibers in the relative pressure (P/P0) range of 0–1. The desorption data of N_2_ isotherm were analyzed by the Barrett–Joyner–Halenda (BJH) method to acquire the value of specific surface area and pore size of the nanofibers. The BET specific surface area of each type of nanofibers was measured for three times, and the average value was reported.

## Results and Discussion

### Optimization of Electrospinning Solutions

Electrospinning solutions with different polymer and precursor concentrations, as listed in Table 1, were prepared and assessed with regard to homogeneity of solutions, uniformity of nanofibers produced, and production yield. First, the required amount of PAN polymer was added to 20 mL of DMF solvent to prepare electrospinning solutions with different polymer concentrations. The electrospinning solutions with 5, 6, 7, and 8 wt % PAN were observed to be electrospinnable. As the solution with 7 wt % PAN resulted in the highest production yield with a problem-free and stable electrospinning process and uniform nanofiber formation, it was chosen for the studies conducted for the determination of precursor concentration.

**Table 1:** List of the Electrospinning Solutions Prepared

electrospinning solutions	PAN concentration (wt %)	salt concentration (PAN/LCC)
S1	5	1:0
S2	6	1:0
S3	7	1:0
S4	8	1:0
S5	7	1:1
S6	7	1:1.5
S7	7	1:2
S8	7	1:3
S9	5	1:1
S10	6	1:1
S11	8	1:1

Afterward, the required amount of precursor salts was added into the 7 w % PAN solution to obtain solutions with 1:1, 1:1.5, 1:2, and 1:3 PAN/LCC ratios in order to determine the maximum salt concentration resulting in uniform and continuous nanofiber formation along with a good yield of LCC nanofibers after the calcination step. A homogeneous nanofiber mat with uniform and continuous nanofibers could be obtained through a stable electrospinning process using a solution containing 1:1 PAN/LCC. When the PAN/LCC ratio was increased to 1:1.5, the stability of the electrospinning process was disturbed, resulting in a nonhomogeneous nanofiber mat formation with some empty areas and polymer particulates on the surface, as shown in Figure 3. Increasing the PAN/LCC ratio further to 1:2 and 1:3 in the electrospinning solutions resulted in dissolution problems, accompanied by coagulation and gelation of the electrospinning solutions, which were attributed to the immiscibility of DMF and water coming from the hydrates of LCC precursors used. Prolonging the dissolution time and applying heat did not resolve these issues, and the resulting solutions could not be used in the electrospinning process.

**Figure 3:**
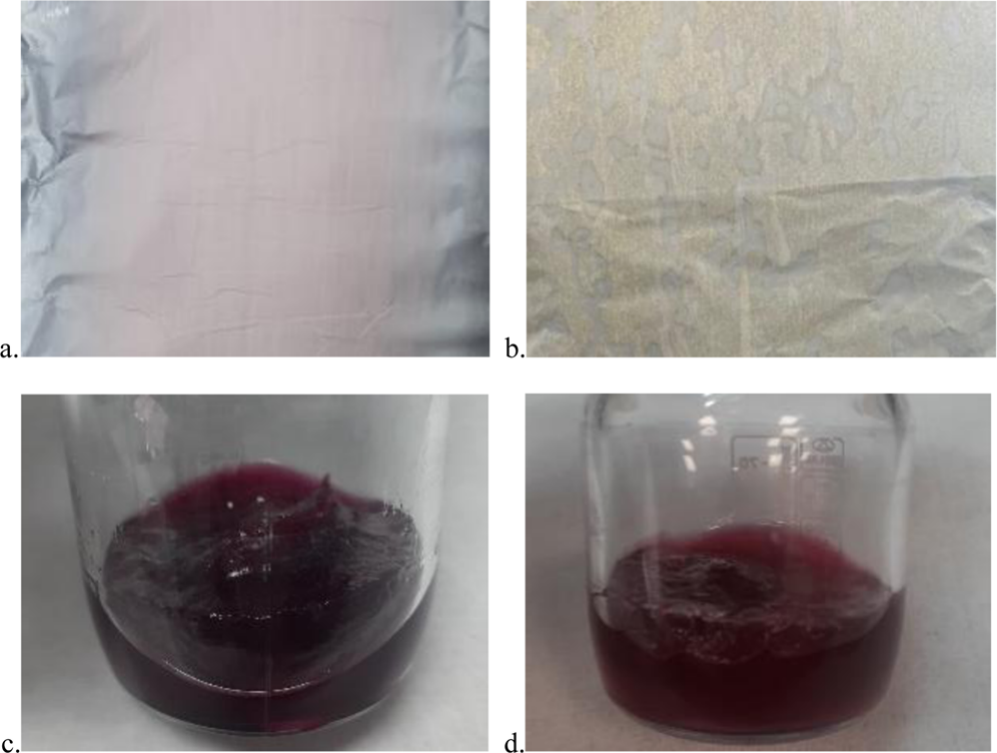
Photographs of nanofiber mats electrospun from electrospinning solutions containing (a) 1:1 and (b) 1:1.5 PAN/LCC and electrospinning solutions containing (c) 1:2 and (d) 1:3 PAN/LCC.

IKA Rotavisc Viscometer and Hanna HI 8633 conductivity meter were used to measure the viscosity and electrical conductivity of the electrospinning solutions with different PAN concentrations (5, 6, 7, and 8 w %) and also their counterparts containing 1:1 PAN/LCC. The results obtained are presented in Table 2. While the viscosity of 5% PAN solution was 503.3 mPa.s, it increased with the increase in the polymer concentration and was measured as 2424 mPa.s for 8% PAN solution. The addition of LCC precursors into the PAN solutions at a PAN/LCC ratio of 1:1 resulted in a decrease in the viscosity of the electrospinning solutions, whereas the electrospinning solutions’ electrical conductivity rose by around 100 folds with the addition of LCC precursors. While the electrical conductivity of prepared PAN solutions was between 0.07 and 0.092 mS cm^–1^, the electrical conductivity for precursor containing solutions was measured between 9.9 and 11 mS cm^–1^.

**Table 2:** Viscosity and Electrical Conductivity of the Electrospinning Solutions

electrospinning solutions	viscosity (mPa.s)	electrical conductivity (mS cm^–1^)
5% PAN	503.3 ± 52.3	0.070 ± 0.0007
6% PAN	949.1 ± 84.0	0.092 ± 0.0001
7% PAN	1661.0 ± 75.4	0.081 ± 0.0002
8% PAN	2424.3 ± 71.4	0.082 ± 0.0003
5% PAN-LCC (1:1)	495.6 ± 47.1	9.90 ± 0.0070
6% PAN-LCC (1:1)	753.7 ± 25.3	10.10 ± 0.0666
7% PAN-LCC (1:1)	1436.6 ± 147.6	10.90 ± 0.0100
8% PAN-LCC (1:1)	2220.0 ± 131.4	11.00 ± 0.0754

### Morphology of Nanofibers

The morphology, average diameter, and homogeneity of nanofibers are important quality aspects as they significantly influence nanofiber performance in targeted applications. In electrospinning, the average fiber diameter is mainly controlled by polymer concentration and solution flow rate, whereas the diameter distribution is governed by a broader range of parameters, including applied voltage, tip-to-collector distance, and the dynamic instabilities associated with jet formation. Deviations from optimal electrospinning conditions promote intermittent jet thinning and thickening, leading to broader diameter distributions even when the average diameter remains unchanged.^45–47^ These effects are expected to be more pronounced in metal–polymer precursor systems due to their higher electrical conductivity.

Although an extensive and systematic optimization of all electrospinning parameters was carried out to achieve a deliberate balance among morphological uniformity (uniform nanofibers with a narrow, near-normal diameter distribution), structural integrity after calcination (continuous nanofibers), and practical material yield, the discussion focuses primarily on the polymer concentration as this parameter was identified as the most critical factor governing stable jet formation and the production of continuous, bead-free nanofiber structures.

The as-electrospun and calcined nanofibers were investigated by SEM in order to study the morphology and size of the fibers. Using ImageJ software, thicknesses of the fibers were measured from at least 50 different distinct locations to analyze the diameter of the nanofibers. These data were then used to obtain mean diameter values, and associated standard deviations were also used to visualize the distribution histogram graphs of produced nanofibers.

SEM images of PAN nanofibers produced from electrospinning solutions with different PAN concentrations and PAN-LCC composite nanofibers, along with the corresponding histograms showing the nanofiber diameter distributions, are presented in Figure 4.

**Figure 4:**
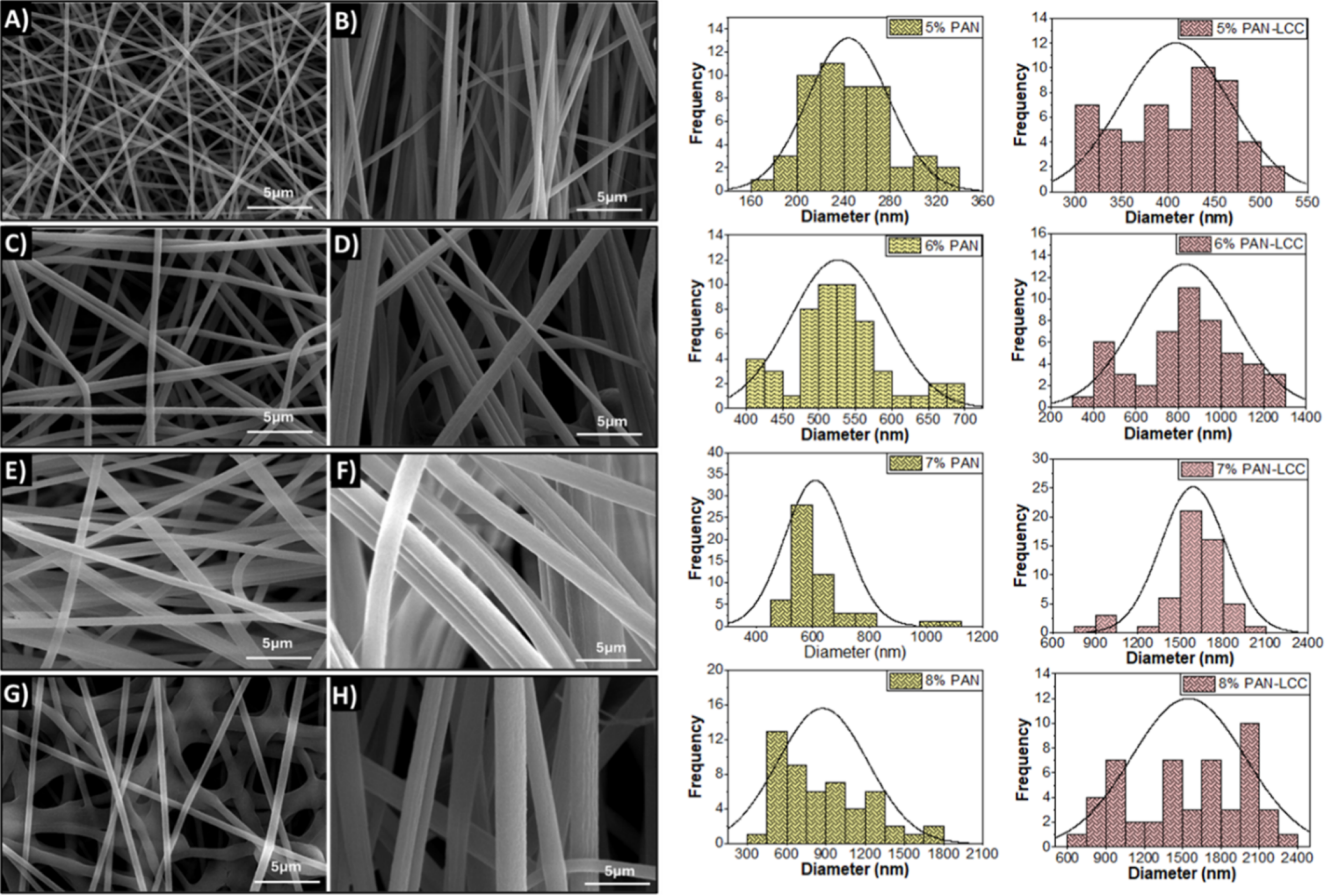
SEM images and corresponding diameter distribution histograms of electrospun nanofibers prepared with different precursor solutions: (A) 5% PAN, (B) 5% PAN-LCC, (C) 6% PAN, (D) 6% PAN-LCC, (E) 7% PAN, (F) 7% PAN-LCC, (G) 8% PAN, and (H) 8% PAN-LCC.

SEM images showed that the nanofibers electrospun from the PAN solutions were uniform and continuous. An increase in the viscosity of the solutions as a result of the increase in PAN concentration resulted in the formation of thicker nanofibers. While the average nanofiber diameter electrospun from 5% PAN solution was 243 nm, it was measured as 882 nm for nanofibers electrospun from solution with 8% PAN concentration, the reasons of which are widely discussed and well-established in the literature. As the viscosity increases, the resistance of fluids to deformation increases, directly influencing jet elongation. Consequently, enhanced viscosity often results in a rise in average fiber diameter.^48^ Nanofibers obtained from 7% PAN solution displayed the most uniform structure with a narrow near-normal distribution.

Addition of the LCC precursors into the PAN solutions affected the solution properties, which consequently influenced both the electrospinning process and the morphology and size of the nanofibers (Figures 4 and 5). The average diameter of the electrospun nanofibers produced from a 5% PAN solution containing LCC precursors at a PAN/LCC ratio of 1:1 was 408 nm, which significantly increased to 1589 nm for the nanofibers electrospun from a 7% PAN solution containing LCC precursors at a PAN/LCC ratio of 1:1. The incorporation of nitrate salts had a dilution effect on the solutions. For all the solutions prepared with different PAN concentrations, a decrease in viscosity was observed accompanied by an increase of 100-fold in electrical conductivity with addition of nitrate salts at a PAN/LCC ratio of 1:1 as a result of which PAN-LCC composite nanofibers showed higher average diameter values than their corresponding pure PAN nanofibers. Though it is frequently observed that adding conductive or nonconductive additives to electrospinning solutions increases the solution’s viscosity, resulting in the formation of thicker nanofibers,^49,50^ studying specifically the PAN solutions in DMF, Qin et al. (2006) observed decrease in solution viscosity with the addition of LiCl, NaCl, CaCl_2_, and NaNO_3_ salts into PAN solutions when the concentration of solutions was more than 4 wt %.^51^ On the other hand, the addition of charge carriers such as conductive filler particles to electrospinning solutions affect not only the viscosity but also the conductivity of the electrospinning solution. Theoretical analyses indicate that increasing the electrical conductivity of the polymer solution enhances the surface charge of the spinning jet, which makes the electrospinning process more stable and fluent, increases the jet flow, and ultimately leads to larger fiber diameters.^51^ The increase in the fiber diameter can also be the effect of surface tension increase in the electrospinning solution due to nitrate salts addition. High surface tension can cause greater resistance to jet expansion, which produces thicker fibers.^52–55^ At 8% PAN concentration, the fiber structure was disturbed, resulting in a loss of uniformity. When the concentration was increased to 8%, thick and nonuniform fibrous structures were observed in the lower layers of the SEM images with finer fiber structures shown up in the upper layers, possibly due to the higher viscosity, blocking the needle and exceeding the optimum level for fiber formation. The average nanofiber diameter was 1544 nm, which is similar to nanofibers electrospun from 7% PAN solutions despite the viscosity increase, possibly due to the formation of thick and thin fibers. The formation of nonhomogeneous nanofiber mat was also reflected on the histogram and standard deviation value, which was nearly doubled, showing the nanofiber diameters distributed in a broader range.

**Figure 5:**
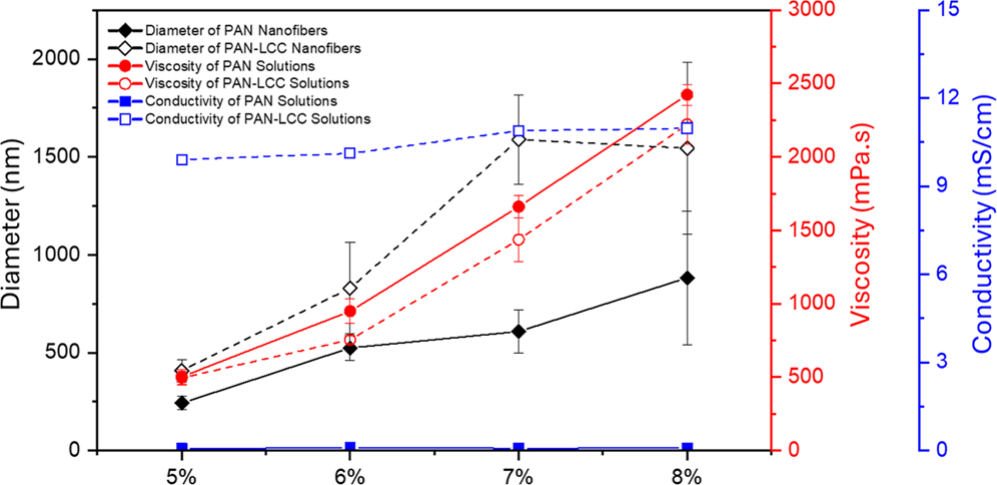
Variations in viscosity and conductivity of electrospinning solutions and corresponding average diameters of pure PAN and PAN-LCC composite nanofibers.

The diameter distribution histograms showed that the 7% PAN and 7% PAN-LCC nanofibers had the most uniform and homogeneous distribution among the produced nanofibers. As a result, due to their more homogeneous distribution and larger production volume than the other samples, the study focused on nanofibers produced from PAN solutions with 7 w % PAN concentration and performed further characterization and measurements on these fibers. From this point on, if anything else is not mentioned, the PAN nanofibers are electrospun from electrospinning solutions containing 7 wt % PAN polymer and PAN-LCC composite nanofibers are electrospun from 7 wt % PAN solutions containing LCC precursors at a PAN/LCC ratio of 1:1.

PAN-LCC composite nanofibers were calcined at three different temperatures as 700, 800, and 900 °C to remove organic matter and achieve the desired perovskite phase. The calcination process was carried out under atmospheric conditions at a heating rate of 4 °C/min. After calcination, the resulting nanofibers were transferred to a carbon tape, coated with gold, and imaged using a scanning electron microscope. It was observed that, during the calcination process the nanofiber morphology was preserved, and uniform and continuous LCC perovskite nanofibers were obtained. Diameter measurements were performed on LCC nanofibers calcined at various temperatures, yielding average diameter values of 578, 576, and 573 nm at calcination temperatures of 700, 800, and 900 °C, respectively (Figure 6). The average nanofiber diameter was calculated as 578 nm for the nanofibers calcined at 700 °C, which was 63.6% smaller than PAN-LCC composite nanofibers owing to the removal of PAN, nitrates, and water from the nanofiber structure with the calcination process. Furthermore, the fact that the ceramic nanofibers had similar average diameters at all calcination temperatures investigated suggested that the calcination process at 700 °C completed the transition to the ceramic phase and removed all organics.

**Figure 6:**
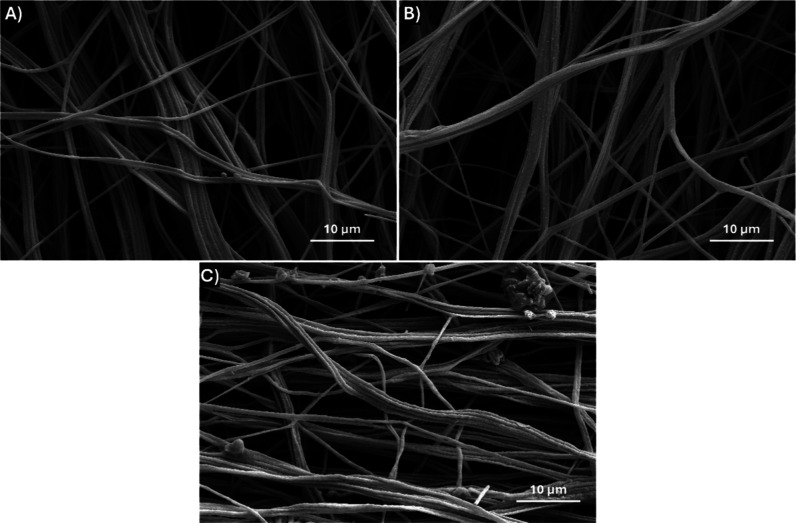
SEM images of LCC perovskite nanofibers after calcination at (A) 700 °C, (B) 800 °C, (C) 900 °C.

### Thermogravimetric Analysis

TGA analysis was performed on pure PAN and PAN-LCC composite nanofibers to investigate their temperature-dependent behavior under atmospheric conditions. Figure 7 depicts the results of the TGA analysis.

**Figure 7:**
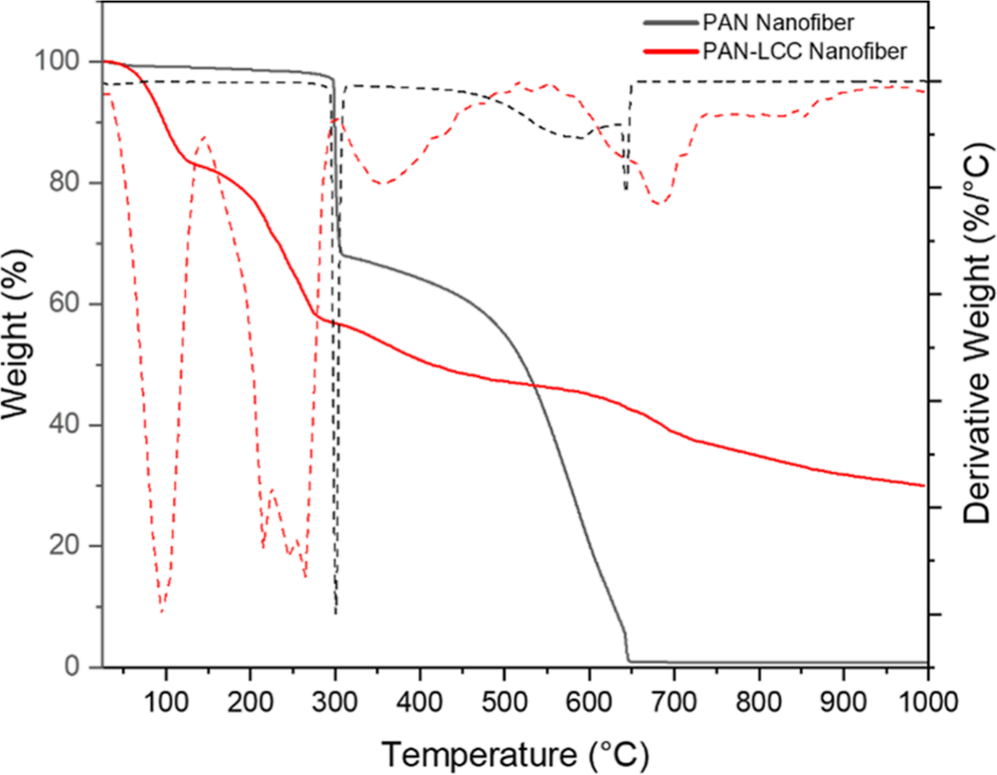
Thermogravimetric analysis of pure PAN and PAN-LCC composite nanofibers.

There were mainly three stages of weight loss for pure PAN nanofibers. An initial minor weight loss is observed below 300 °C, which is attributed to the evaporation of moisture or residual solvent. A major weight loss of around ∼ 29.8% was observed at 300 °C, corresponding to the cyclization of the nitrile groups in the PAN and charring of the PAN backbone.^56–61^ Thereafter a continuous weight loss was observed with a drastic decrease in weight between 500 and 650 °C, showing that carbonized PAN and other organics were decomposed and removed away from the system. According to the TGA curve of PAN nanofibers, the decrease in weight of PAN nanofibers continued until 650 °C, after which almost no residues were left.

For the PAN-LCC nanofiber samples, the initial weight loss around the 25 and 150 °C (∼18.6%) was accounted to the release of hydration water from the metal nitrates, and the evaporation of physically adsorbed moisture. Between 200 and 300 °C, the weight loss of around ∼24.8% was caused by the thermal breakdown of nitrates, which released nitrogen oxides and formed intermediate oxide species. Following this decomposition step, there was a slight decomposition (∼16.8%) occurred over a broader range, from around 300 to 600 °C, which involved the cyclization of the nitrile groups in the PAN and charring of the PAN backbone. Between 600 and 900 °C, the carbonized PAN and other organics, which corresponded to about 13% of the initial weight, were decomposed and removed away from the system. The cyclization and decomposition of the PAN polymer were observed to occur at higher temperatures compared to pure PAN, likely due to interactions between the metal species and the polymer, as well as the higher thermal conductivity of the metals, which delays heat transfer within the system and consequently shifts the thermal decomposition of PAN to higher temperatures. Notably, the final residual mass of about 30% indicated the formation of thermally stable LCC perovskite phases.

To evaluate whether the behavior observed in the TGA analysis reflects actual calcination conditions, additional calcination experiments were performed at 700 and 900 °C in a furnace while monitoring weight changes. Only a minor difference of approximately 1% in weight loss was observed between the two temperatures, indicating that calcination at 700 °C already resulted in a largely stabilized nanofiber structure. The final residual mass values were measured as 15.29% and 14.02% for samples calcined at 700 and 900 °C, respectively. These findings suggest the gradual weight loss observed in the TGA of PAN-LCC nanofibers above 700 °C is attributed to kinetic effects arising from the rapid heating rates and short exposure time at elevated temperatures typically used in TGA experiments, rather than ongoing chemical decomposition. Therefore, TGA data should be interpreted with caution when correlating with practical thermal treatment conditions. Nevertheless, previous studies have reported the formation of a stable structure at temperatures above 500 °C.^37^

### X-ray Diffraction Analysis

In order to determine the crystal structure, phase content, and orientation of the obtained materials, X-ray diffraction (XRD) analysis was performed. In this context, PAN-LCC composite nanofibers electrospun from an electrospinning solution with 7 w % PAN concentration, containing 1:1 PAN/LCC and LCC nanofibers obtained by calcination at 700, 800, and 900 °C, for 3 h were analyzed. The obtained results are presented in Figure 8.

**Figure 8:**
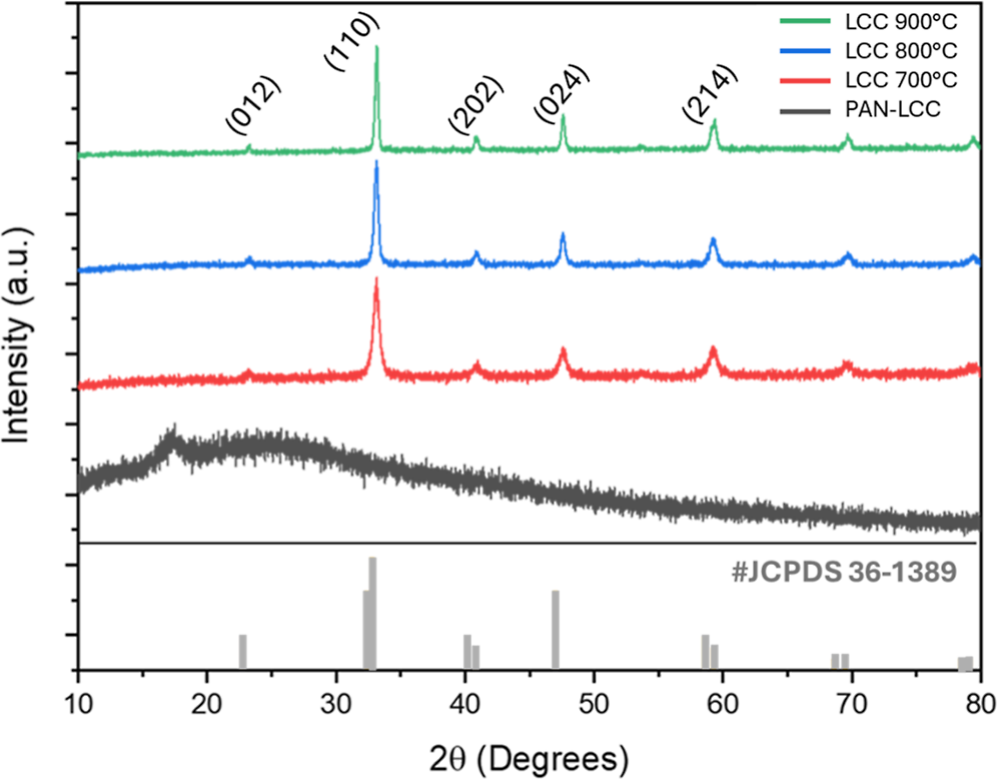
XRD curves of PAN-LCC composite nanofibers and LCC perovskite nanofibers prepared by calcination at 700, 800, and 900 °C.

The calcination temperature plays a key role in determining the morphology, phase purity, crystallinity, and specific surface area of the perovskite nanofibers. Higher temperatures generally enhance crystallinity and phase purity but can reduce the surface area and compromise the continuity of the nanofiber morphology, whereas lower temperatures may lead to incomplete phase formation.^62,63^ In the present work, the calcination parameters were primarily optimized to ensure single-phase formation and continuous nanofiber morphology, which are essential prerequisites for any subsequent application-oriented evaluation.

Peaks were observed at approximately 16.8° and 24.9°, corresponding to the semicrystalline structure of the PAN nanofibers and relaxed amorphous structure, respectively.^64,65^ In contrast, samples exposed to calcination showed the elimination of PAN polymers from the system, as well as a clear crystallization beginning and progressive development of the perovskite ceramic structure with the oxidation of nitrate salts via heat treatment. The XRD patterns of nanofibers after calcination confirmed the single-phase formation of LCC nanofibers. Phase identification confirmed that the calcined nanofibers crystallized into a rhombohedral perovskite phase, consistent with the JCPDS-36–1389 reference pattern. The calcination at 700 °C was found to provide both single-phase LCC formation and continuous nanofiber structure. When the temperature of the calcination process was raised from 700 °C to 800 and 900 °C, it was observed that the prominent (110) peak became narrower proving increase in crystallization and crystal size. Additionally, the crystallite sizes for the LCC perovskite nanofibers prepared were determined by XRD measurements utilizing the Scherrer equation based on a full-width at half maxima (FWHM) of the (110) plane and the crystallite size values are listed in Table 3. While the average crystallite size of LCC nanofibers calcined at 700 °C was calculated as 15.74 nm, the crystallite sizes grew up to 33.92 nm with increasing calcination temperature up to 900 °C. Furthermore, an increase in calcination temperature corresponded to enhanced crystallinity and partial intensification of grain growth. This phenomenon is attributable to the complete volatilization of the polymeric constituents at elevated temperatures, facilitating the nucleation and growth of ceramic nanofiber crystallites. Calcination at 900 °C, while increasing crystal size and crystallinity as indicated by XRD, resulted in surface segregation as observed in XPS analyses and likely reduced surface area due to grain coarsening.

**Table 3:** FWHM and Crystallite Size Calculation by the Scherrer Equation

calcination temperature	peak degree (2θ)	fwhm (2θ)	crystallite size (nm)
700 °C	33.09	0.553	15.74
800 °C	33.10	0.351	24.62
900 °C	33.15	0.255	33.92

However, it should be noted that the crystallite sizes obtained using the Scherrer equation represent apparent values as the method assumes that peak broadening arises solely from finite crystallite size and neglects microstrain contributions. To further evaluate this aspect, Williamson–Hall (W–H) analysis was performed. The resulting plots exhibited low linear correlation coefficients (*R* ^2^) (Supporting Information Figure S1), indicating that the assumption of isotropic strain is not valid for the present system. This also implies that the crystallite size values obtained from the Scherrer equation may include strain contributions and should therefore be considered as approximate. Nevertheless, the Scherrer results are still useful for comparative purposes.

Taken together, the XRD results, supported by complementary analyses, indicate that all three calcination temperatures lead to the formation of single-phase LCC nanofibers. However, considering both structural integrity and economic efficiency, 700 °C can be regarded as the minimum effective temperature for achieving single-phase formation with a continuous nanofiber morphology. These findings highlight the critical role of thermal treatment in controlling the crystal structure, morphology, and stability of LCC nanofibers, in good agreement with previous reports on perovskite nanofiber systems.^36,66^

### Fourier-Transform Infrared Spectroscopy Analysis

FTIR analysis was carried out to identify the structural alterations in the nanofiber structure. Figure 9 shows the FTIR spectra recorded in the spectral range 600–4000 cm^–1^ of the pure PAN nanofibers, PAN-LCC composite nanofibers, and LCC perovskite nanofibers.

**Figure 9:**
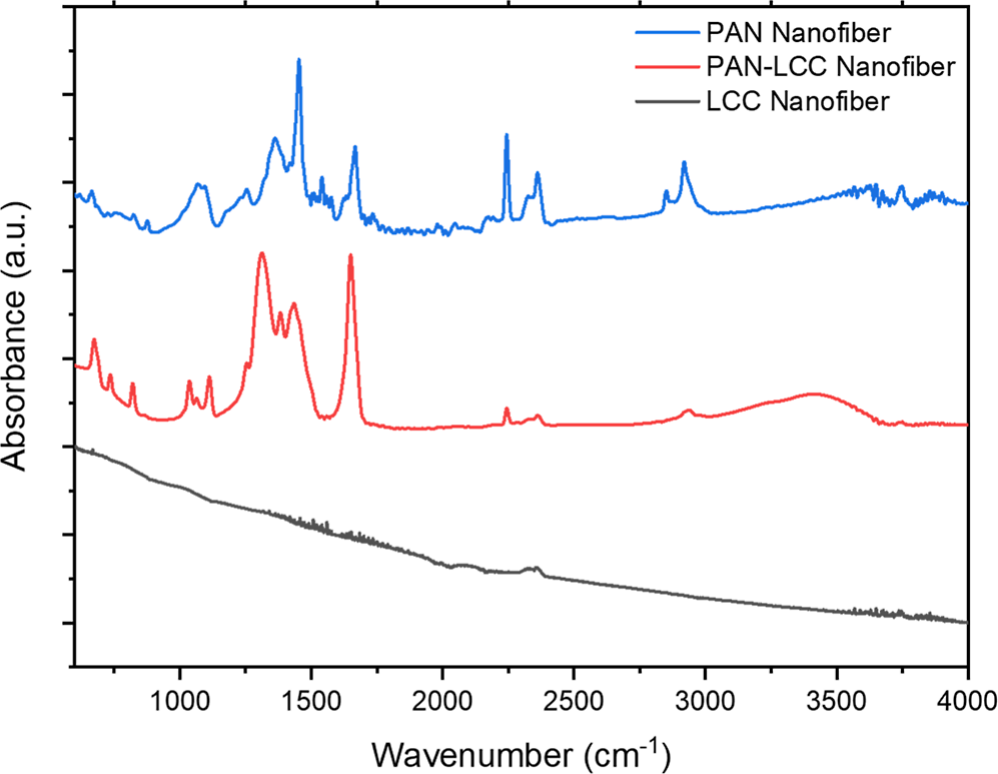
FTIR spectra of pure PAN nanofibers, PAN-LCC composite nanofibers, and LCC perovskite nanofibers obtained by calcination at 700 °C.

The FTIR spectra provided an accurate representation of the chemical changes that took place with addition of metallic salts and calcination. The spectrum of PAN nanofibers is distinguished by a significant C≡N stretching band about 2243 cm^–1^, in addition to other peaks associated with the polymer’s C–H vibrations. The peaks in the spectra of PAN nanofibers can be assigned as follows: 3626 cm^–1^ (OH stretching), 2918 and 2850 cm^–1^ (C–H asymmetric and symmetric stretchings in CH, CH_2_, and CH_3_ groups), 2242 cm^–1^ (C≡N stretching), 1666 cm^–1^ (C=C stretching), 1452 cm^–1^ (CH_3_ bending and CH_2_ scissor vibration), 1362 cm^–1^ (CH_3_ symmetric bending vibration in C–CH_3_), 1253 cm^–1^ (C–N stretching), and 1070–1040 cm^–1^ (C=N bending).^61,67–73^ Incorporating nitrate salts into PAN nanofibers resulted in a large absorption peak about 3400 cm^–1^ along with two lower intensity infrared absorption band located at 1650 cm^–1^, which are assigned to the stretching and bending vibrational modes of physically adsorbed H_2_O molecules.^74,75^ Nitrates displayed characteristic peaks for a mixture of N–O stretching and bending of N=O between 1430 and 1310 cm^–1^, plus a sharp peak at 819 cm^–1^ for NO_3_^–^, which are in well agreement with the reported literature.^76^ The bands related with the PAN polymer were also observed with less intensity due to the dominant peaks corresponding to the hydrated nitrate salts. Upon calcination at 700 °C, the spectrum underwent a notable change: all PAN-related bands disappeared, indicating the polymer’s complete removal. The results of FTIR analysis were in good agreement with XRD and TGA results. The results showed that PAN could be used as a template to create ceramic nanofibers in a controlled thermal process.^77–80^

### X-ray Photoelectron Spectroscopy Analysis

For applications such as gas sensing and solid oxide fuel cells, surface oxygen vacancies play a critical role as the oxygen reduction reaction (ORR) primarily occurs at or near the surface. Consequently, surface defect chemistry is often more directly correlated with functional performance than bulk composition.^81,82^ In addition, owing to the one-dimensional nanofiber morphology, the surface-to-volume ratio of the present materials is substantially higher than that of conventional bulk or thin-film counterparts. As a result, the surface region probed by XPS represents a significant fraction of the material’s electrochemically active volume, thereby providing valuable insight into the materials’ potential catalytic behavior.^63^

Changes in surface chemistry and elemental ratios were analyzed using X-ray photoelectron spectroscopy both before and after the calcination process. The XPS spectra obtained from PAN/LCC composite nanofibers and LCC perovskite nanofibers prepared by calcination at 700, 800, and 900 °C, respectively, are presented in Figure 10, and the atomic concentrations are presented in Table 4.

**Figure 10:**
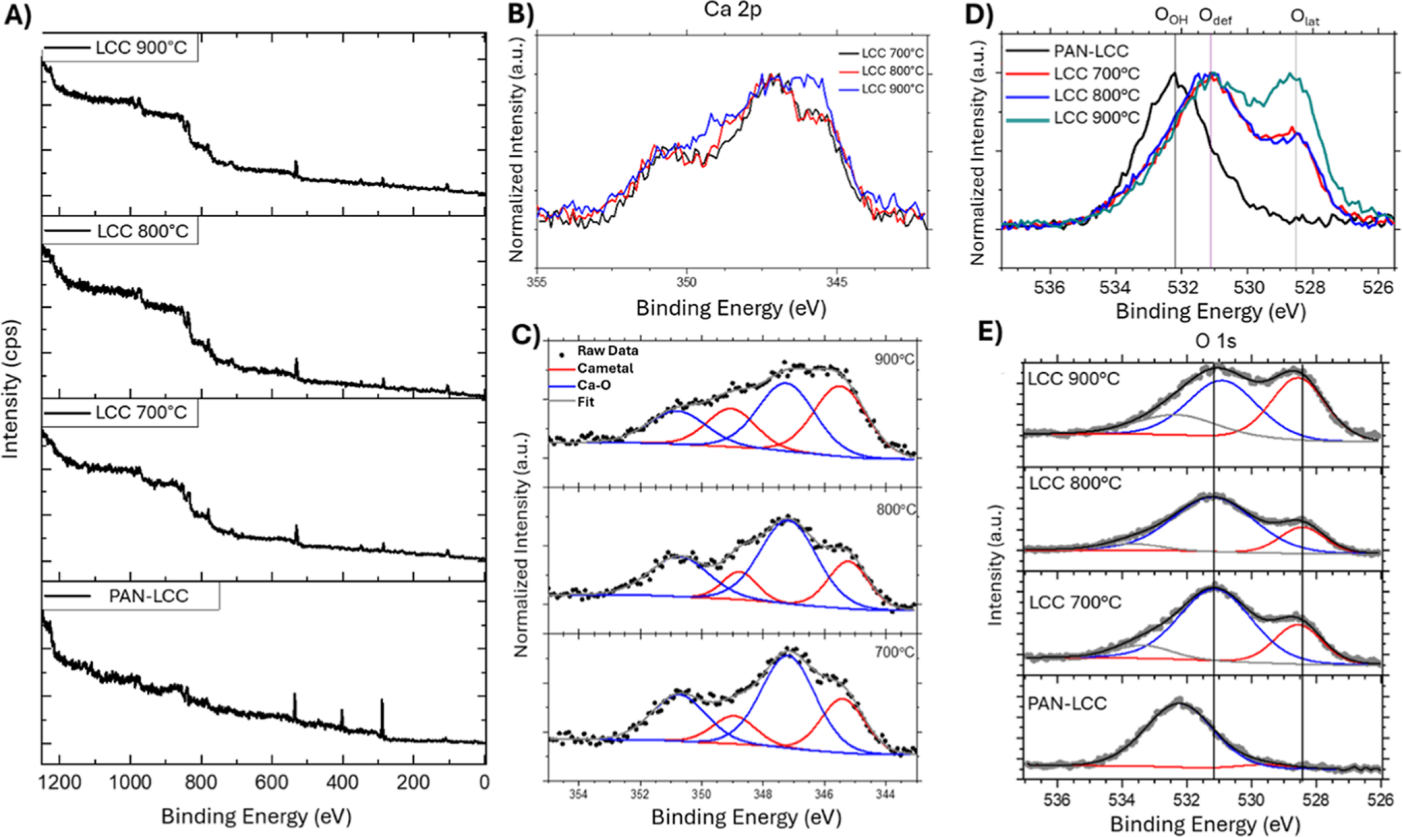
XPS spectra of nanofiber samples (A) survey, (B) Ca 2p fittings, (C) Ca distribution, (D) O 1s fittings, (E) oxygen distribution.

**Table 4:** Atomic Concentration, Oxygen Distribution, and Ca Distribution Ratios of PAN-LCC Composite Nanofibers and LCC Perovskite Nanofibers Obtained by Calcination at Different Temperatures along with Oxygen Components and Calcium Compositions

	atomic concentrations				oxygen distribution			Ca distribution	
samples	La (%)	Ca (%)	Co (%)	O (%)	O_lat_ (%)	O_def_ (%)	O_OH_ (%)	Ca-metal (%)	Ca–O (%)
PAN-LCC	6.21	2.84	4.94	85.99	-	5.21	94.79	-	-
LCC 700 °C	6.99	10.98	6.09	75.93	24.02	66.71	9.27	30.0	70.0
LCC 800 °C	7.82	9.66	6.21	76.29	23.28	68.16	8.56	28.6	71.4
LCC 900 °C	6.85	11.61	6.89	74.63	35.66	56.96	7.38	49.2	50.8

The XPS spectra showed that the calcination process mostly removed the carbon peak (C 1s) from organic matter at 288 eV, as well as the N 1s (≈400 eV) peak, which was visible due to DMF, PAN, and metal salts in the fiber structure. Given the elemental ratios, the expected La/Ca ratio for calcined nanofiber samples was 0.6/0.4 = 1.5; however, this ratio was calculated to be 0.63 for fibers calcined at 700 °C. As the temperature rose to 900 °C, the La/Ca ratio decreased to 0.59. At the same time, the (La + Ca)/Co ratio, which was expected to be 1 based on the prepared stoichiometry, was found to be 2.95 in nanofibers calcined at 700 °C. This is assumed to be the result of strong Ca segregation during the calcination process, as well as the presence of Ca-rich regions on the surface (Figure 8). This segregation is fundamentally driven by the difference in ionic radius between the La host atoms and the Ca dopant atoms.^83^ This mismatch induces lattice stress, which combined with elevated temperatures, promotes segregation. However, it is important to note that the ionic radius of Ca^2+^ (1.34 Å) is closer to that of La^3+^ (1.36 Å) compared to other common dopants like Sr^2+^ (1.44 Å). This closer size match reduces the lattice stress and, consequently, mitigates the tendency for segregation.^36,84^ Furthermore, as the temperature increased, the Ca-metal/Ca–O ratio also increased (Figure 10 b,c, Table 4). However, XRD graphs show that the expected LCC phase structure was acquired, with no CoO or Co_3_O_4_ phases. It was concluded that the segregation occurred solely in the oxygen-rich surface regions throughout the calcination process, while the desired LCC phase persisted in the bulk material.

The concentration distributions of oxygen components were determined from O 1s spectral deconvolution (Figure 10e) and are given in Table 4. The fit analysis revealed a high proportion of surface absorbed water on the PAN-LCC composite nanofibers. Upon calcination, the intensity of oxygen component related to hydroxide (O_OH_) near the surface decreased, while both the oxygen content within the lattice (O_lat_) and vacancy sites (O_def_) increased between the temperatures of 700 and 800 °C. However, when the temperature rose to 900 °C, the O_lat_ level increased further from ∼24% at 700–800 °C to ∼36%, while the O_def_ fraction decreased from ∼67% to ∼57%. This trend suggest that higher temperatures promote oxygen incorporation into the lattice and reduce vacancy concentrations, likely due to enhanced diffusion, segregation, and structural reorganization at elevated temperatures.^56^ However, because the concentration of oxygen vacancies and highly reactive surface oxygen diminishes above 800 °C, sintering temperatures exceeding 800 °C may be unfavorable for applications that rely on high vacancy densities or surface reactivity.^56,85^

### Brunauer–Emmett–Teller Analysis

The surface areas of pure PAN nanofibers, PAN-LCC composite nanofibers, and LCC perovskite nanofibers obtained by calcination at 700 °C were determined based on the nitrogen adsorption–desorption isotherms using the BET method. For the BET measurement, the prepared samples were heated for approximately 8 h before being degassed. Following adsorption with N_2_ gas, the prepared samples were tested.

Figure 11 presents the N_2_ isotherms which are type IV (IUPAC Classification) with a H3 hysteresis loop in the high-pressure range (0.8 < *P*/*P*_o_ > 1), which can be associated with the existence of a mesoporous structure generally pore size ranging from 2 to 50 nm.^86–88^ The adsorption increased with the increase in the surface area.

**Figure 11:**
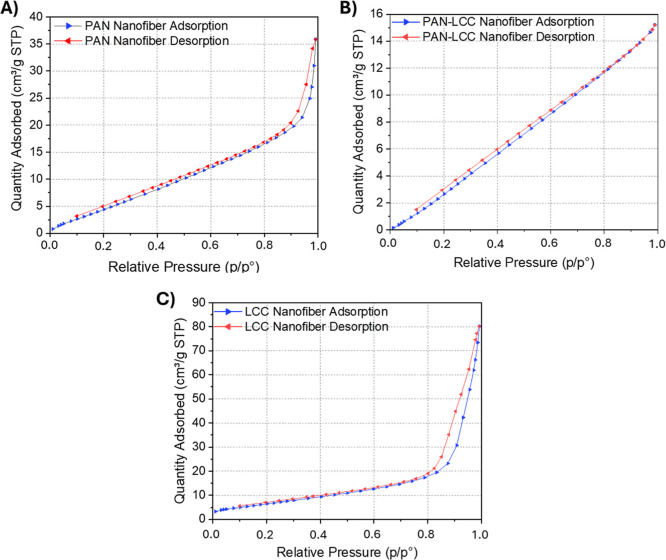
Nitrogen adsorption–desorption isotherms of (A) pure PAN nanofibers, (B) PAN-LCC composite nanofibers, and (C) LCC perovskite nanofibers.

Figure 12 shows the variations in surface area and pore size with the addition of precursor salts and the calcination process. The surface area for the PAN nanofiber samples was determined as 19.86 m^2^ g^–1^, whereas the surface area for the PAN-LCC composite nanofibers was calculated as 14.94 m^2^ g^–1^, resulting in about a 25% drop on average surface area, which was likely caused by the increase in fiber diameter with the addition of LCC precursors into the electrospinning solution. However, ceramic LCC nanofibers obtained from PAN-LCC composite nanofibers by the calcination process had an average surface area of 20.13 m^2^ g^–1^ due to the decrease in average nanofiber diameter with calcination and the surface of the nanofibers becoming rough after the calcination. The values obtained were much higher than the values Shingange et al. reported for LCO nanofibers.^40^ Meanwhile, the average pore size increased nearly 3-fold, rising from 5.92 nm in PAN-LCC composite nanofibers to 15.97 nm in LCC perovskite nanofibers, and is thought to be due to the reduction in nanofiber diameter from 1589 to 578 nm during the calcination process, which resulted in larger gaps between the fibers.

**Figure 12:**
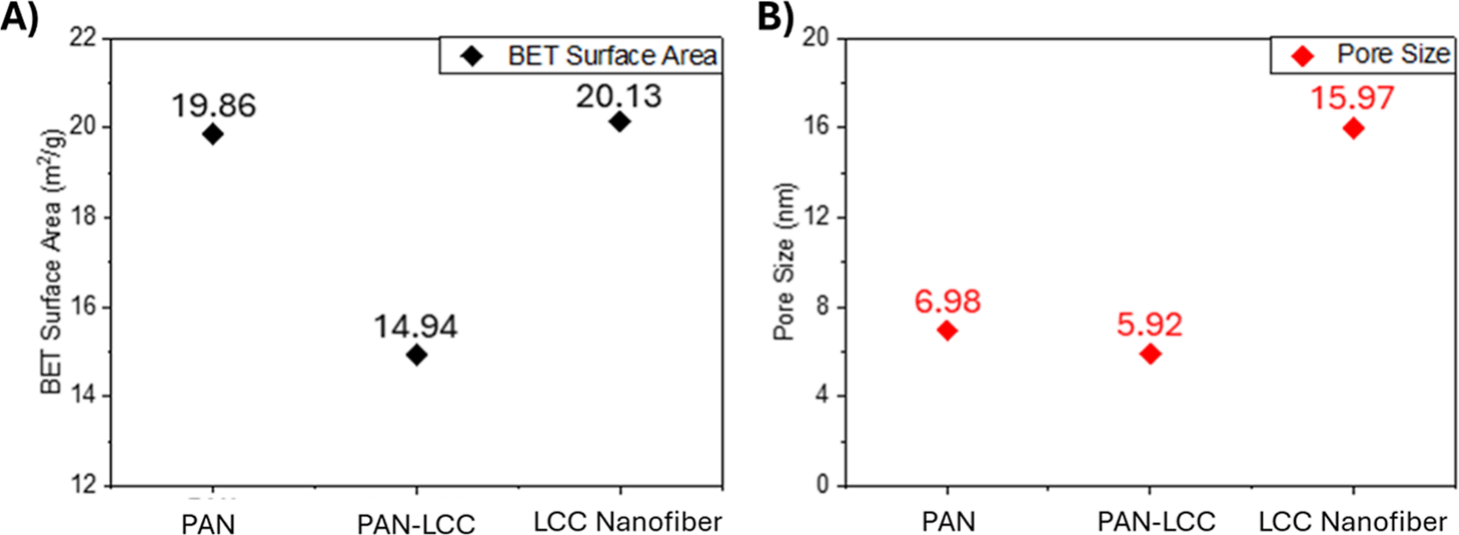
BET analysis of PAN, PAN-LCC, and LCC nanofibers. (A) Surface area, (B) pore size values.

Additionally, an analysis of the cumulative data revealed a notable increase in the pore volume within LCC nanofibers due to the calcination process, accompanied by an increase in the pore size (Figure 13). This is further supported by the fact that LCC nanofibers demonstrated superior adsorption values relative to PAN and PAN-LCC nanofibers, as shown in Figure 11.

**Figure 13:**
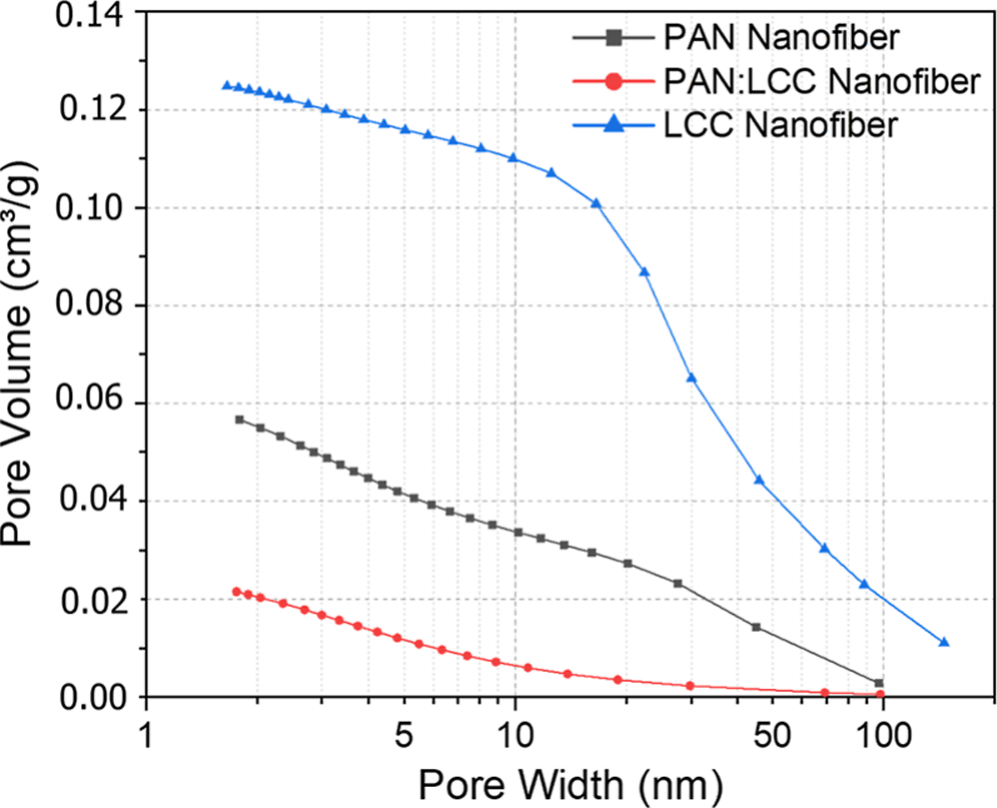
Pore volume vs pore width comparison of PAN nanofibers, PAN-LCC composite nanofibers, and LCC perovskite nanofibers.

## Conclusions

La_0.6_Ca_0.4_CoO_3_ (LCC) perovskite nanofibers were successfully fabricated by the electrospinning technique using PAN as a carrier polymer. For all the solutions prepared with different PAN concentrations, a decrease in viscosity was observed accompanied by an increase of 100 fold in electrical conductivity with addition of nitrate salts at a PAN/LCC ratio of 1:1. Consequently, the PAN-LCC composite nanofibers exhibited larger average diameters compared to the corresponding pure PAN nanofibers. The nanofibers obtained from the electrospinning solution with 7% PAN concentration containing LCC precursors at a PAN/LCC ratio of 1:1 displayed the most uniform nanofiber structure and diameter distribution. Thermogravimetric experiments showed that water, organic compounds, nitrates in the composite fibers were mostly volatilized, and pure LCC nanofibers were obtained at around 700 °C. FTIR also confirmed the complete removal of organics upon calcination at 700 °C. The average diameter of LCC nanofibers after the calcination process was measured as 584 nm. XRD verified that the calcined nanofibers crystallized into a rhombohedral perovskite structure and clearly showed that the annealing affected their structural properties. The average crystallite size increased from 15.74 nm at 700 °C to 33.92 nm at 900 °C, confirming enhanced crystallinity and the partial intensification of grain growth. XPS analysis revealed that surface oxygen vacancy concentrations varied with calcination temperature, and significant Ca segregation occurred at elevated temperatures. Although the desired phase was obtained in the bulk structure, XPS analysis revealed deviations from the expected La + Ca stoichiometry, with Ca-rich regions observed at the surface. In addition, the BET investigation showed an increase in pore size in nanofiber structures after calcination, as well as a considerable increase in surface area when compared to PAN-LCC nanofibers. The results demonstrate that La_0_._6_Ca_0_._4_CoO_3_ (LCC) perovskite nanofibers with nanoscale diameters, high-surface area, tunable oxygen vacancy concentrations, one-dimensional morphology, and well-defined crystal structures can be successfully synthesized using the proposed approach. These physicochemical characteristics establish a robust structural and compositional foundation for potential applications in intermediate-temperature solid oxide fuel cells, energy conversion devices, catalysis, and gas sensing. While morphological stability after high-temperature calcination is demonstrated, comprehensive evaluation of long-term thermal stability and device-level performance under realistic operating conditions remains the focus of future work.

## Supplementary Material



## Notes

Williamson–Hall (W–H) plots prepared using XRD data for LCC nanofibers calcined at 700, 800, and 900 °C (PDF)

## References

[1] Kizildag, N. Recent Advances in Applications of Ceramic Nanofibers. In Nanofibers: Synthesis, Properties and Applications; Kumar, B. , Ed.; IntechOpen eBooks, 2021; pp 1–26.

[2] Aksan O. A., Kizildag N. (2025). Electrospun Nanofiber-Based
Perovskite Cathodes for
Solid Oxide Fuel Cells: A review. Energy Fuels.

[3] Pascariu P., Homocianu M. (2019). ZnO-Based
Ceramic Nanofibers: Preparation, Properties
and Applications. Ceram. Int..

[4] Liu Y., Ding Y., Zhang L., Gao P.-X., Lei Y. (2012). CeO_2_ Nanofibers for in
Situ O_2_ and CO Sensing in Harsh Environments. RSC Adv..

[5] Han T., Ma S. Y., Xu X. L., Xu X. H., Pei S. T., Tie Y., Cao P. F., Liu W. W., Wang B. J., Zhang R., Zhang J. L. (2020). Rough SmFeO_3_ Nanofibers as an Optimization
Ethylene Glycol Gas Sensor Prepared by Electrospinning. Mater. Lett..

[6] Ma L., Ma S. Y., Shen X. F., Wang T. T., Jiang X. H., Chen Q., Qiang Z., Yang H. M., Chen H. (2018). PrFeO_3_ Hollow Nanofibers
as a Highly Efficient Gas Sensor for Acetone
Detection. Sens Actuators B Chem..

[7] George, G. ; Senthil, T. ; Luo, Z. ; Anandhan, S. Sol-gel Electrospinning of Diverse Ceramic Nanofibers and Their Potential Applications. In Electrospun Polymers and Composites: Ultrafine Materials, High Performance Fibres and Wearables; Dong, Y. , Baji, A. , Seeram, R. , Eds.; Elsevier eBooks, 2020; pp 689–764.

[8] Mercante L. A., Andre R. S., Mattoso L. H. C., Correa D. S. (2019). Electrospun Ceramic
Nanofibers and Hybrid-Nanofiber Composites for Gas Sensing. ACS Appl. Nano Mater..

[9] Zhang J., Zhang X., Wang L., Zhang J., Liu R., Sun Q., Ye X., Ma X. (2023). Fabrication and Applications of Ceramic-Based
Nanofiber Materials Service in High-Temperature Harsh Conditions–A
Review. Gels.

[10] Liu Z., Gu Y., Bi L. (2023). Applications of Electrospun Nanofibers in Solid Oxide
Fuel Cells – A Review. J. Alloys Compd..

[11] Gao D., Zhao R., Yang X., Chen F., Ning X. (2021). Bicomponent
PLA Nanofiber Nonwovens as Highly Efficient Filtration Media for Particulate
Pollutants and Pathogens. Membranes.

[12] Sanna C., Zhang W., Costamagna P., Holtappels P. (2021). Synthesis
and Electrochemical Characterization of La_0.6_Sr_0.4_Co_0.2_Fe_0.8_O_3−δ_/Ce_0.9_Gd_0.1_O_1.95_ Co-Electrospun Nanofiber
Cathodes for Intermediate-Temperature Solid Oxide Fuel Cells. Int. J. Hydrogen Energy.

[13] Oğlakcıoğlu N., Akduman C., Sarı B. (2021). Investigation of Thermal Comfort
Properties of Electrospun Thermoplastic Polyurethane Fiber Coated
Knitted Fabrics for Wind-resistant Clothing. Polym. Eng. Sci..

[14] Fennessey S. F., Farris R. J. (2004). Fabrication of Aligned and Molecularly Oriented Electrospun
Polyacrylonitrile Nanofibers and the Mechanical Behavior of Their
Twisted Yarns. Polymer.

[15] Ramaseshan R., Sundarrajan S., Jose R., Ramakrishna S. (2007). Nanostructured
Ceramics by Electrospinning. J. Appl. Phys..

[16] Chronakis I. S. (2005). Novel Nanocomposites
and Nanoceramics Based on Polymer Nanofibers Using Electrospinning
Process–A Review. J. Mater. Process.
Technol.

[17] Sigmund W., Yuh J., Park H., Maneeratana V., Pyrgiotakis G., Daga A., Taylor J., Nino J. C. (2006). Processing and Structure
Relationships in Electrospinning of Ceramic Fiber Systems. J. Am. Ceram. Soc..

[18] Nalbandian M. J., Zhang M., Sanchez J., Choa Y.-H., Nam J., Cwiertny D. M., Myung N. V. (2016). Synthesis
and Optimization of Fe_2_O_3_ Nanofibers for Chromate
Adsorption from Contaminated
Water Sources. Chemosphere.

[19] Nalbandian M. J., Greenstein K. E., Shuai D., Zhang M., Choa Y.-H., Parkin G. F., Myung N. V., Cwiertny D. M. (2015). Tailored Synthesis
of Photoactive TiO_2_ Nanofibers and Au/TiO_2_ Nanofiber
Composites: Structure and Reactivity Optimization for Water Treatment
Applications. Environ. Sci. Technol..

[20] Teli M. D., Nadathur G. T. (2020). Reversible Colourimetric
Sensing of Volatile Phase
by Dye Doped Electrospun Silica Based Nanofibers. J. Environ. Chem. Eng..

[21] Zhang W., Wang X., Zhang Q., Wang L., Xu Z., Li Y., Huang S. (2020). Li 7 La_3_Zr_2_O_12_ Ceramic
Nanofiber-Incorporated Solid Polymer Electrolytes for Flexible Lithium
Batteries. ACS Appl. Energy Mater..

[22] Zaidi S. D. A., Wang C., Shao Q., Gao J., Zhu S., Yuan H., Chen J. (2020). Polymer-Free Electrospun
Separator
Film Comprising Silica Nanofibers and Alumina Nanoparticles for Li-Ion
Full Cell. J. Energy Chem..

[23] Xue J., Xie J., Liu W., Xia Y. (2017). Electrospun Nanofibers:
New Concepts,
Materials, and Applications. Acc. Chem. Res..

[24] Panda P. K. (2007). Ceramic
Nanofibers by Electrospinning Technique–A Review. Trans. Indian Ceram. Soc..

[25] Esfahani H., Jose R., Ramakrishna S. (2017). Electrospun
Ceramic Nanofiber Mats
Today: Synthesis, Properties, and Applications. Materials.

[26] Nagarajan S., Belaid H., Pochat-Bohatier C., Teyssier C., Iatsunskyi I., Coy E., Balme S., Cornu D., Miele P., Kalkura N. S., Cavaillès V., Bechelany M. (2017). Design of Boron Nitride/Gelatin Electrospun
Nanofibers for Bone Tissue Engineering. ACS
Appl. Mater. Interfaces.

[27] Du Z., Zhou X., Ye P., Zeng X., Gan C. L. (2020). Shape-Memory
Actuation in Aligned Zirconia Nanofibers for Artificial Muscle Applications
at Elevated Temperatures. ACS Appl. Nano Mater..

[28] Jiang W., Loh H., Low B. Q. L., Zhu H., Low J., Heng J. Z. X., Tang K. Y., Li Z., Loh X. J., Ye E., Xiong Y. (2023). Role of Oxygen Vacancy
in Metal Oxides for Photocatalytic CO_2_ Reduction. Appl. Catal., B.

[29] Parwaiz S., Jennings J. R., Harunsani M. H., Khan M. M. (2025). Recent Advances
in LaCoO_3_ -Based Perovskite Nanostructures for Electrocatalytic
and Photocatalytic Applications. Crit. Rev.
Solid State Mater. Sci..

[30] Guo X., Sun X., Wang L., Qiao Y., Dong S. (2025). Investigation of the
Structure and Electrochemical Performance of Perovskite Oxide La_1–x_Ca_x_CrO_3_ Utilized as Electrode
Materials for Supercapacitors. Coatings.

[31] Murai K.-I., Kori S., Nakai S., Moriga T. (2018). Effect of
Thermoelectric
Material of Ca or Fe-Doped LaCoO_3_. Int. J. Mod. Phys. B.

[32] Ateia E. E., Mohamed A. T., Morsy M. (2019). Humidity Sensor Applications Based
on Mesopores LaCoO_3_. J. Mater. Sci.:Mater.
Electron..

[33] Wang Y., Guo X., Du D., Yang S. (2022). Study of Ce, Ca, Fe, and Mn-Doped
LaCoO_3_ Perovskite Oxide for the Four-Way Purification of
PM, NO_x_, CO, and HC from Diesel Engine Exhaust. Materials.

[34] Moorthi K., Sivakumar B., Chokkiah B., Valdes H., Mohan S. (2024). Morphological
Impact of Perovskite-Structured Lanthanum Cobalt Oxide (LaCoO_3_) Nanoflakes Toward Supercapacitor Applications. ACS Appl. Nano Mater..

[35] Sun X., Pei Z., Guo X., Ye X., Wang L., Zhang Y., Dong S. (2025). Impact of Ca Ions Substitution at A-Site on LaCoO_3_ Perovskite
Energy Applications. Materials Science and Engineering:
B.

[36] Sezer M., Öztoprak I. ˙., Ahsen A. S. ¸., Oral A. Y., Büyükaksoy A. (2020). Correlation
of Phase,
Microstructure and Surface Chemistry Evolution to the Long-Term Performance
Stability of (La, Ca)­CoO_3_ SOFC Electrodes. J. Electrochem. Soc..

[37] Wang, J. ; Dong, X. ; Qu, Z. ; Liu, G. ; Yu, W. Fabrication and Characterization of LaCoO_3_ Nanofibers via an Electrospinning Technique. Int. J. Chem. 2010, 2(1), 161 –167. 10.5539/ijc.v2n1p161.

[38] Dong B., Li Z., Li Z., Xu X., Song M., Zheng W., Wang C., Al-Deyab S. S., El-Newehy M. (2010). Highly Efficient
LaCoO_3_ Nanofibers Catalysts for Photocatalytic Degradation
of Rhodamine B. J. Am. Ceram. Soc..

[39] Shim J., Lopez K. J., Sun H.-J., Park G., An J.-C., Eom S., Shimpalee S., Weidner J. W. (2015). Preparation and Characterization
of Electrospun LaCoO_3_ Fibers for Oxygen Reduction and Evolution
in Rechargeable Zn–Air Batteries. J.
Appl. Electrochem..

[40] Shingange K., Swart H. C., Mhlongo G. H. (2020). Design of Porous
P-Type LaCoO_3_ Nanofibers with Remarkable Response and Selectivity
to Ethanol
at Low Operating Temperature. Sens Actuators
B Chem..

[41] Wahed F., Shah S. S., Hayat K., Shah S. K., Aziz Md. A. (2022). Conduction
Mechanisms and Thermoelectric Applications of La_1‑x_Sr_x_CoO_3_ Nanofibers. J.
Mater. Sci..

[42] Guo X., Qiu C., Wang L., Ding J., Zhang J., Wan H., Guan G. (2024). The A-Site and B-Site Regulation of LaCoO_3_ Nanofiber Based
on Electrospinning for Boosting Photocatalytic CO_2_ Conversion. J. Photochem. Photobiol. A Chem..

[43] Guo X., Tian Z., Qu J., Ye X., Wang L., Zhang Y., Dong S., Ju H. (2025). Enhanced the Electrochemical
Performance in Sr-Doped LaCoO_3_ Nanofibers. J. Alloys Compd..

[44] Cao J., Liu L., Han B., Wang Z., Li B., Demir M., Ma P. (2025). Tailoring
LaCoO_3_ Perovskite Oxides via Ce Substitution
and Nanofiber Architecture for Enhanced Electrochemical Storage Performance. ACS Appl. Energy Mater..

[45] Sencadas V., Correia D. M., Areias A., Botelho G., Fonseca A. M., Neves I. C., Gomez Ribelles J. L., Lanceros Mendez S. (2012). Determination
of the Parameters Affecting Electrospun Chitosan Fiber Size Distribution
and Morphology. Carbohydr. Polym..

[46] Sencadas V., Ribeiro C., Nunes-Pereira J., Correia V., Lanceros-Méndez S. (2012). Fiber Average
Size and Distribution Dependence on the Electrospinning Parameters
of Poly­(Vinylidene Fluoride–Trifluoroethylene) Membranes for
Biomedical Applications. Appl. Phys. A: Mater.
Sci. Process..

[47] Jacobs V., Anandjiwala R. D., Maaza M. (2010). The Influence of Electrospinning
Parameters on the Structural Morphology and Diameter of Electrospun
Nanofibers. J. Appl. Polym. Sci..

[48] Munawar M. A., Nilsson F., Schubert D. W. (2025). Tunable
Diameter of Electrospun Fibers
Using Empirical Scaling Laws of Electrospinning Parameters. Mater. Chem. Phys..

[49] Al
Saif Y., Cselkó R. (2025). Revolutionizing Electrospinning:
A Review of Alternating Current and Pulsed Voltage Techniques for
Nanofiber Production. Processes.

[50] Zuo W., Zhu M., Yang W., Yu H., Chen Y., Zhang Y. (2005). Experimental
Study on Relationship between Jet Instability and Formation of Beaded
Fibers during Electrospinning. Polym. Eng. Sci..

[51] Qin X., Yang E., Li N., Wang S. (2007). Effect of Different
Salts on Electrospinning of Polyacrylonitrile (PAN) Polymer Solution. J. Appl. Polym. Sci..

[52] Doustvandi B., Imani R., Yousefzadeh M. (2024). Study of Electrospun PVDF/GO Nanofibers
as a Conductive Piezoelectric Heart Patch for Potential Support of
Myocardial Regeneration. Macromol. Mater. Eng..

[53] Costa E. L., Muniz E. C., Cava C. E. (2023). PEDOT:PSS/AgNWs
Nanofibers Obtained
by Electrospun and the Post-Treatment via DMSO Vapor Exposure. Synth. Met..

[54] Mpukuta O., Dincer K., Özaytekin I. (2020). Effect of
Dynamic Viscosity on Nanofiber
Diameters and Electrical Conductivity of Polyacrylonitrile Nanofibers
Doped Nano-Cu Particles. International Journal
of Innovative Engineering Applications.

[55] Adabavazeh Z., Johari N., Baino F. (2025). Electrospun Conductive Polymer Scaffolds:
Tailoring Fiber Diameter and Electrical Properties for Tissue Engineering
Applications. Mater. Today Commun..

[56] Zheng F., Qian Y., Pang S. (2023). Promoting the Segregation
of Sr^2+^ from the Perovskite Oxygen Catalyst La_0.5_Sr_0.5_Co_3−δ_ via Quenching. Coatings.

[57] Taheran M., Naghdi M., Brar S. K., Knystautas E., Verma M., Surampalli R. Y., Valero J. R. (2016). Development of Adsorptive
Membranes by Confinement of Activated Biochar into Electrospun Nanofibers. Beilstein J. Nanotechnol..

[58] Nadirah B. N., Ong C. C., Saheed M. S. M., Yusof Y. M., Shukur M. F. (2020). Structural
and Conductivity Studies of Polyacrylonitrile/Methylcellulose Blend
Based Electrolytes Embedded with Lithium Iodide. Int. J. Hydrogen Energy.

[59] Xue T. J., McKinney M. A., Wilkie C. A. (1997). The Thermal
Degradation of Polyacrylonitrile. Polym. Degrad.
Stab..

[60] Henrici-Oliv, G. ; Oliv, S. Inter-versus Intramolecular Oligomerization of Nitrile Groups in Polyacrylonitrile. J. Polym. Sci. 1981, 5(8), 457 –461. 10.1007/BF00255983.

[61] Moafi H. F., Fallah Shojaie A., Ali Zanjanchi M. (2011). Photoactive Polyacrylonitrile Fibers
Coated by Nano-Sized Titanium Dioxide: Synthesis, Characterization,
Thermal Investigation. J. Chil. Chem. Soc..

[62] Park J.-W., Shin J., Ju Y.-W. (2023). Influence
of Calcination Temperature
on Electrochemical Properties of Perovskite Oxide Nanofiber Catalysts. Energies.

[63] Enrico A., Zhang W., Lund Traulsen M., Sala E. M., Costamagna P., Holtappels P. (2018). La_0.6_Sr_0.4_Co_0.2_Fe_0.8_O_3‑δ_ Nanofiber Cathode for Intermediate-Temperature
Solid Oxide Fuel Cells by Water-Based Sol-Gel Electrospinning: Synthesis
and Electrochemical Behaviour. J. Eur. Ceram.
Soc..

[64] Liu X. M. N. P., Jamil A., Lee D., Song J. (2025). Development of Self-Healing
Nanofiber-Reinforced Green Composites via Dual-Nozzle Coaxial Electrospinning
for Enhanced Mechanical Performance and Damage Recovery. Polym. Eng. Sci..

[65] Ryšánek P., Benada O., Tokarský J., Syrový M., Čapková P., Pavlík J. (2019). Specific Structure,
Morphology, and Properties of Polyacrylonitrile (PAN) Membranes Prepared
by Needleless Electrospinning; Forming Hollow Fibers. Materials Science and Engineering: C.

[66] Kumar D. A., Selvasekarapandian S., Nithya H., Leiro J., Masuda Y., Kim S.-D., Woo S.-K. (2013). Effect of Calcium Doping on LaCoO_3_ Prepared by Pechini Method. Powder
Technol..

[67] Mathur R. B., Bahl O. P., Sivaram P. (1992). Thermal Degradation
of Polyacrylonitrile
Fibres. Curr. Sci..

[68] Simitzis J. (1994). Modified Polyacrylonitrile
for Adsorption Applications. Acta Polym..

[69] De P., Sathyanarayana D. N., Sadasivamurthy P., Sridhar S. (2001). Synthesis, Structural
Characterization, Thermal Studies and Chain Dynamics of Poly­(methacrylonitrile
peroxide) by NMR Spectroscopy. Polymer.

[70] De P., Sathyanarayana D. N., Sadasivamurthy P., Sridhar S. (2002). Reactivity Ratios for
the Oxidative Copolymerizations of Indene with Methyl Methacrylate
and Methacrylonitrile. Eur. Polym. J..

[71] Zhang D., Karki A. B., Rutman D., Young D. P., Wang A., Cocke D., Ho T. H., Guo Z. (2009). Electrospun Polyacrylonitrile
Nanocomposite Fibers Reinforced with Fe_3_O_4_ Nanoparticles:
Fabrication and Property Analysis. Polymer.

[72] Minagawa M., Miyano K., Takahashi M., Yoshii F. (1988). Infrared Characteristic
Absorption Bands of Highly Isotactic Poly­(Acrylonitrile). Macromolecules.

[73] Deng S., Bai R., Chen J. P. (2003). Behaviors and Mechanisms of Copper Adsorption on Hydrolyzed
Polyacrylonitrile Fibers. J. Colloid Interface
Sci..

[74] Iorio Y. D., Aguirre M. E., Brusa M. A., Grela M. A. (2012). Surface Chemistry
Determines Electron Storage Capabilities in Alcoholic Sols of Titanium
Dioxide Nanoparticles. A Combined FTIR and Room Temperature EPR Investigation. J. Phys. Chem. C.

[75] Kholief M. G., Hesham A. E.-L., Hashem F. S., Mohamed F. M. (2024). Synthesis and Utilization
of Titanium Dioxide Nano Particle (TiO_2_NPs) for Photocatalytic
Degradation of Organics. Sci. Rep.

[76] Gupta N., Kumar A., Dhawan S. K., Dhasmana H., Kumar A., Kumar V., Verma A., Jain V. K. (2020). Metal Nanoparticles
Enhanced Thermophysical Properties of Phase Change Material for Thermal
Energy Storage. Mater. Today: Proc..

[77] Tas S., Kaynan O., Ozden-Yenigun E., Nijmeijer K. (2016). Polyacrylonitrile
(PAN)/Crown Ether Composite Nanofibers for the Selective Adsorption
of Cations. RSC Adv..

[78] Naraghi, M. ; Chasiotis, I. Mechanics of PAN Nanofibers. In Major Accomplishments in Composite Materials and Sandwich Structures; Springer Netherlands: Dordrecht, 2009; pp 757–778.

[79] Parsafard N., Aghajari G. (2024). La-Supported SnO_2_–CaO Composite Catalysts
for Efficient Malachite Green Degradation under UV–Vis Light. BMC Chem..

[80] Khan W. S. (2017). Chemical
and Thermal Investigations of Electrospun Polyacrylonitrile Nanofibers
Incorporated with Various Nanoscale Inclusions. Journal of Thermal Engineering.

[81] Boukamp B. A., Carru J.-C. (2024). Characterization
of Porous La_0.6_Sr_0.4_Co_0.8_Fe_0.2_O_3‑δ_ Based
Cathode Films for Intermediate Temperature Solid Oxide Fuel Cells.
An Electrochemical Impedance Study. Solid State
Ion.

[82] Siebenhofer M., Riedl C., Schmid A., Limbeck A., Opitz A. K., Fleig J., Kubicek M. (2022). Investigating
Oxygen Reduction Pathways
on Pristine SOFC Cathode Surfaces by *in Situ* PLD
Impedance Spectroscopy. J. Mater. Chem. A Mater..

[83] Nikolaeva O., Kapishnikov A., Gerasimov E. (2021). Structural Insight into La_0.5_Ca_0.5_Mn_0.5_Co_0.5_O_3_ Decomposition
in the Methane Combustion Process. Nanomaterials.

[84] Sezer M., Sezer H., Ahsen A. S. ¸., Büyükaksoy A. (2023). Surface Chemistry
and Phase Evolution in Dense Sr- or Ca-Doped LaCoO_3_ Ceramics
and Their Correlation to Surface Exchange and Chemical Diffusion Coefficients. J. Phys. Chem. C.

[85] Whitten A., Guo D., Tezel E., Denecke R., Nikolla E., McEwen J.-S. (2024). Deconvoluting
XPS Spectra of La-Containing Perovskites from First-Principles. JACS Au.

[86] Zhang N., Ruan S., Yin Y., Li F., Wen S., Chen Y. (2018). Self-Sacrificial Template-Driven LaFeO_3_/α-Fe_2_O_3_ Porous Nano-Octahedrons for Acetone Sensing. ACS Appl. Nano Mater..

[87] Thirumalairajan S., Girija K., Hebalkar N. Y., Mangalaraj D., Viswanathan C., Ponpandian N. (2013). Shape Evolution of Perovskite LaFeO_3_ Nanostructures: A Systematic Investigation of Growth Mechanism,
Properties and Morphology Dependent Photocatalytic Activities. RSC Adv..

[88] Lee J., Yoon S., Hyeon T., Oh S. M., Kim K. B. (1999). Synthesis
of a New Mesoporous Carbon and Its Application to Electrochemical
Double-Layer Capacitors. Chem. Commun..

