# Supplementary material for: La0.6Ca0.4CoO3 Perovskite Nanofibers by Electrospinning: Preparation and Properties
Source: ACS Omega. 2026 May 19;11(21):30786–800. doi: 10.1021/acsomega.5c13104 (PMC13234647; doi:10.1021/acsomega.5c13104)
Supplement: Supplementary file 1 [file ao5c13104_si_001.pdf]

.

# $La_{0.6}Ca_{0.4}CoO_3$ perovskite nanofibers by electrospinning: Preparation and properties


Onur Alp AKSAN[1], Esin Cagla KONUKCU[1], Mehmet SEZER[2],
Leyla ÇOLAKEROL ARSLAN[3], Nuray KIZILDAG[1,*]

[1]Institute of Nanotechnology, Gebze Technical University, Kocaeli, 41400, Turkey.
[2]Department of Materials Science and Engineering, Gebze Technical University, Kocaeli, 41400, Turkey
[3]Department of Physics, Gebze Technical University, Kocaeli, 41400, Turkey

*Corresponding author: nuraykizildag@gtu.edu.tr

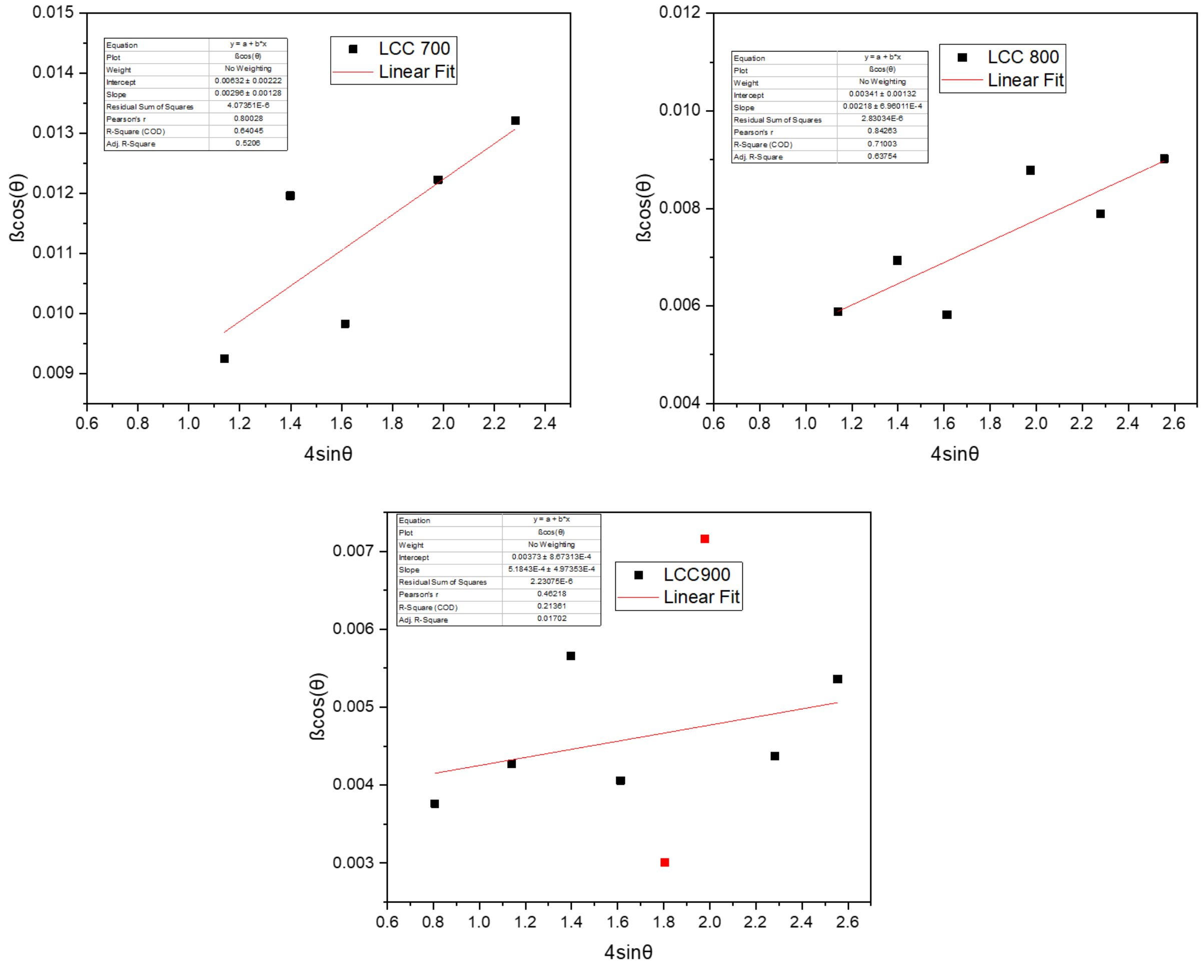


Figure S1. Williamson–Hall (W–H) plots prepared using XRD data for LCC nanofibers calcined at 700, 800 and 900°C.

It should be noted that the crystallite sizes obtained using the Scherrer equation represent apparent values, as the method assumes that peak broadening arises solely from finite crystallite size and neglects microstrain contributions. To further evaluate this aspect, Williamson–Hall (W–H) analysis was performed. The resulting plots exhibited low linear correlation coefficients ($R^2$) (Figure S1), indicating that the assumption of isotropic strain is not valid for the present system. This also implies that the crystallite size values obtained from the Scherrer equation may include strain contributions and should therefore be considered as approximate. Nevertheless, the Scherrer results are still useful for comparative purposes.
